# A Dendrimer Peptide (KK2DP7) Delivery System with Dual Functions of Lymph Node Targeting and Immune Adjuvants as a General Strategy for Cancer Immunotherapy

**DOI:** 10.1002/advs.202300116

**Published:** 2023-03-22

**Authors:** Rui Zhang, Lin Tang, Yusi Wang, Yaomei Tian, Siwen Wu, Bailing Zhou, Chunyan Dong, Binyan Zhao, Yuling Yang, Daoyuan Xie, Li Yang

**Affiliations:** ^1^ State Key Laboratory of Biotherapy and Cancer Center West China Hospital Sichuan University and Collaborative Innovation Center for Biotherapy Chengdu 610041 China

**Keywords:** delivery system, dendrimer peptide, immune adjuvants, KK2DP7, lymph node targeting

## Abstract

The clinical efficacy of personalized cancer vaccines still needs to be improved due to their insufficient immune effect. The development of innovative adjuvants and lymph node‐targeted delivery systems is the key to improving the clinical efficacy of personalized vaccines. However, there is still a lack of an adjuvant delivery system that is simple in preparation and capable of mass production and integrates adjuvant and lymph node targeted delivery functions. Here, this work reports that a simple dendrimer polypeptide (KK2DP7) nanoparticle enhances the immune efficacy of an OVA/neoantigen‐based vaccine. Due to its multiple functions as a delivery vehicle, immune adjuvant, and facilitator of dendritic cell migration, KK2DP7 efficiently increases the efficiency of antigen uptake and cross‐presentation by antigen‐presenting cells (APCs) and delivers antigens to lymph nodes via APCs. Strikingly, the antitumor effect of KK2DP7/OVA is superior to that of commonly used adjuvants such as poly(I:C), CpG, and aluminum adjuvant combined with OVA. Furthermore, KK2DP7/OVA combined with anti‐PD‐1 antibody is able to prevent tumor recurrence in a postoperative recurrent tumor model. Thus, KK2DP7‐based cancer vaccines alone or in combination with immune checkpoint blockade therapies to treat tumors or postoperative tumor recurrence are a powerful strategy to enhance antitumor immunity.

## Graphical abstract

KK2DP7 in combination with OVA can form nanovaccine that can target lymph nodes after subcutaneous injection. As a delivery vehicle, it can efficiently deliver OVA to DCs via caveolin‐ and clathrin‐dependent pathways. As an immune adjuvant, it can stimulate DC maturation by activating the TLR2‐NF‐*κ*B signaling pathway, increasing the efficiency of DC cross‐presentation to antigens and inducing subsequent immune responses.

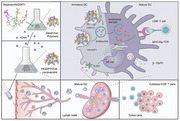

## 1 Introduction

Tumor vaccines have the characteristics of high specificity, low toxicity, and few side effects. They are good choices for replacing or assisting traditional therapy to enhance efficacy. Improving the in vivo immune stimulation effect of vaccines is an important research direction in the field of tumor immunotherapy. Adaptive immune responses are primarily initiated in secondary lymphoid organs, and efficient accumulation of vaccines in lymph nodes (LNs) is a prerequisite for cancer vaccines to induce robust antigen‐specific immune responses.^[^ ^1^ ^]^ LNs are the main site of antigen presentation, where a large number of APCs are present. These cells are adjacent to the initial T cells and can achieve rapid antigen presentation after antigen uptake. Therefore, the effective delivery of tumor vaccines to LNs is an important strategy to improve the efficacy of vaccines. At present, the effective delivery of tumor vaccines to LNs mostly depends on nanodelivery systems.^[^ ^2^ ^]^ Because nanotechnology has the inherent characteristic of being captured by APCs, the use of nanotechnology for cancer vaccine design shows great promise. However, current nanovaccine systems still have obstacles in achieving effective tumor treatment. This may be partly due to the unsatisfactory design of vaccine vectors, mainly due to their single function. Most vectors do not have the efficacy of immune adjuvants, and their complex synthesis process is not conducive to subsequent large‐scale use.^[^ ^2b,c^ ^]^ Some studies have reported that some new delivery systems can be used as both delivery systems and adjuvants and can also target lymph nodes. However, the synthesis process is still complex, which is not conducive to quality control and large‐scale preparation in the transformation research process.^[^ ^3^ ^]^ Therefore, it is urgent to develop a new multifunctional delivery system that is simple to prepare and integrates immune adjuvant and lymph node‐specific targeted delivery functions.

**Scheme 1:**
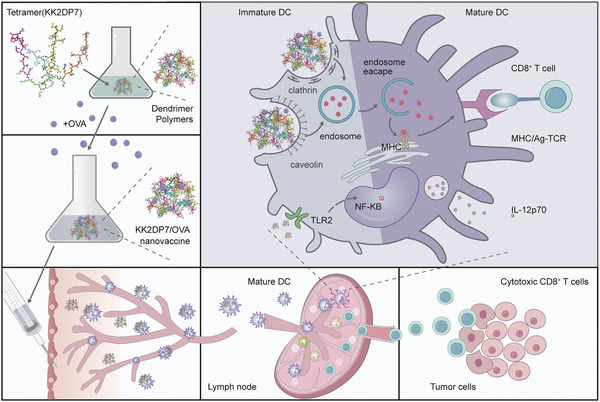
Schematic illustration of a multifunctional nanovaccine with lymph node targeting as a general strategy for cancer immunotherapy. The dendritic peptide KK2DP7 can form a dendrimer polymer in aqueous solution, which in combination with OVA can form a nanovaccine that can target lymph nodes via DC transport after subcutaneous injection. As a delivery vehicle, it can efficiently deliver OVA to DCs via caveolin‐ and clathrin‐dependent pathways with efficient endosomal escape. As an immune adjuvant, it can stimulate DC maturation by activating the TLR2‐NF‐*κ*B signaling pathway, increasing the efficiency of DC cross‐presentation to antigens and inducing subsequent immune responses.

Herein, we designed a simple dendrimer nanoparticle system (KK2DP7) as a novel cancer therapeutic nanovaccine to enhance the immune efficacy of OVA‐based vaccines. DP7 (VQWRIRVAVIRK) is a novel cationic hydrophilic antimicrobial peptide that was developed based on the amino acid activity prediction method in our previous study. We then modified DP7 with cholesterol and found that DP7‐C has dual functions as a delivery vehicle and an immune adjuvant.^[^ ^4^ ^]^ Although previous experiments have demonstrated that DP7‐C can be used to enhance the antitumor effect of antigen‐loaded DC vaccines, due to the complex and time‐consuming preparation of dendritic cell (DC) vaccines in vitro, we believe that direct incubation of DP7‐C with antigens for administration will allow patients to benefit from vaccines more quickly. Unfortunately, there was no obvious LN targeting effect after DP7‐C was complexed with antigens. Therefore, in this experiment, we designed a dendrimer KK2DP7 polymer nanovaccine system with enhanced LN targeting. As a delivery vehicle, it can efficiently deliver OVA to BMDCs via caveolin‐ and clathrin‐dependent pathways. As an immune adjuvant, it can stimulate BMDC maturation by activating the TLR2‐NF‐*κ*B signaling pathway. In addition, the entry of KK2DP7/OVA into LNs is achieved by the transport of DCs (**Scheme** **1**). In animal experiments, the antitumor effect of KK2DP7/OVA was superior to that of commonly used adjuvants such as poly(I:C), CpG, and aluminum adjuvant combined with OVA. Furthermore, KK2DP7/OVA combined with anti‐PD‐1 antibody showed excellent antitumor effects and was able to prevent tumor recurrence in a postoperative recurrent tumor model, and KK2DP7/neoantigen combined with anti‐PD‐1 antibody also showed excellent antitumor effects in the LL2 tumor model. Thus, KK2DP7‐based cancer vaccines alone or in combination with immune checkpoint blockade (ICB) therapies to treat tumors are a powerful general strategy to enhance antitumor immunity.

## 2 Results

### 2.1 Characterization of KK2DP7/OVA and Evaluation of Its LN Targeting

**Figure 1:**
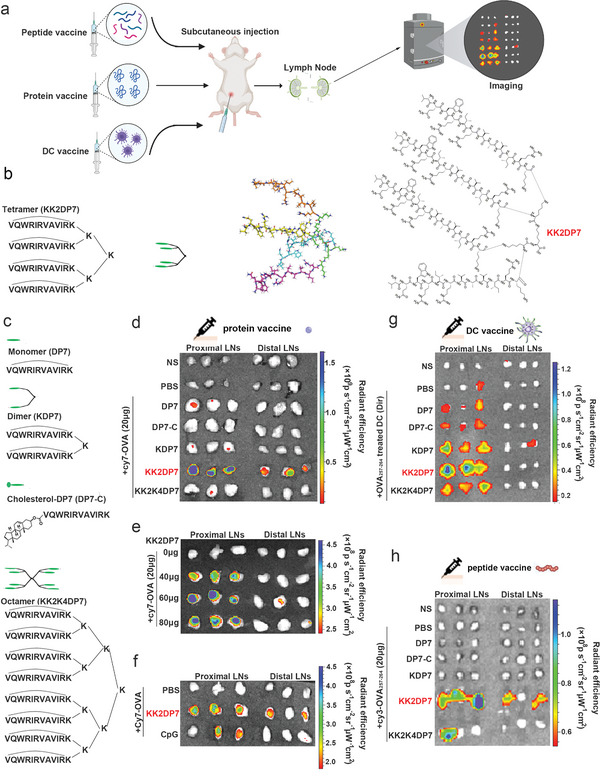
Evaluation of lymph node targeting of the KK2DP7 combination peptide vaccine, protein vaccine and DC vaccine. a) The experimental flow diagram of this study. b) Sequences and structures of KK2DP7. c) Sequences and structures of DP7, KDP7, KK2K4DP7, and DP7‐C. d) In vivo spectrum imaging system (IVIS) fluorescence imaging of isolated inguinal LNs from C57BL/6 mice (*n* = 3) at 4 h after DP7 series (60 µg per mouse)/Cy7‐OVA (20 µg per mouse) administration. e) IVIS fluorescence imaging of isolated inguinal LNs from C57BL/6 mice (*n* = 3) 24 h after administration of different concentrations of KK2DP7/Cy7‐OVA (20 µg per mouse). f) IVIS fluorescence imaging of isolated inguinal LNs from C57BL/6 mice (*n* = 3) 24 h after administration of KK2DP7 (60 µg per mouse)/Cy7‐OVA (20 µg per mouse) or CpG (40 µg per mouse)/Cy7‐OVA (20 µg per mouse). g) IVIS fluorescence imaging of isolated inguinal LNs from C57BL/6 mice (*n* = 3) 24 h after administration of DP7 series (25 µg mL^−1^)/OVA_257–264_ (10 µg mL^−1^)‐treated Dir‐labeled DCs. h) IVIS fluorescence imaging of isolated inguinal LNs from C57BL/6 mice (*n* = 3) 4 h after DP7 series (60 µg per mouse)/Cy3‐OVA_257–264_ (20 µg per mouse) administration.

Efficient delivery of antigens and adjuvants to LNs is ideal for vaccine design.^[^ ^5^ ^]^ We first verified the LN targeting of the designed DP7 peptides combined with OVA protein, DP7 peptides combined with OVA_257–264_ peptide and BMDCs loaded with the DP7 peptide/OVA_257–264_ complex (**Figure** **1**a). Among all designed DP7 series groups (Figure 1b–c, Figure S1a,b, Supporting Information), only the KK2DP7‐related groups showed the most significant increase in the fluorescence intensity of LNs (Figure 1d,h, Figure S1e–i, Supporting Information). In addition, the fluorescence intensity in the KK2DP7/OVA group was significantly higher than that in the CpG/OVA group (Figure 1f). The above results indicate that the LN targeting of KK2DP7 combined with OVA is excellent. Thus, we conducted a follow‐up study on KK2DP7.

**Figure 2:**
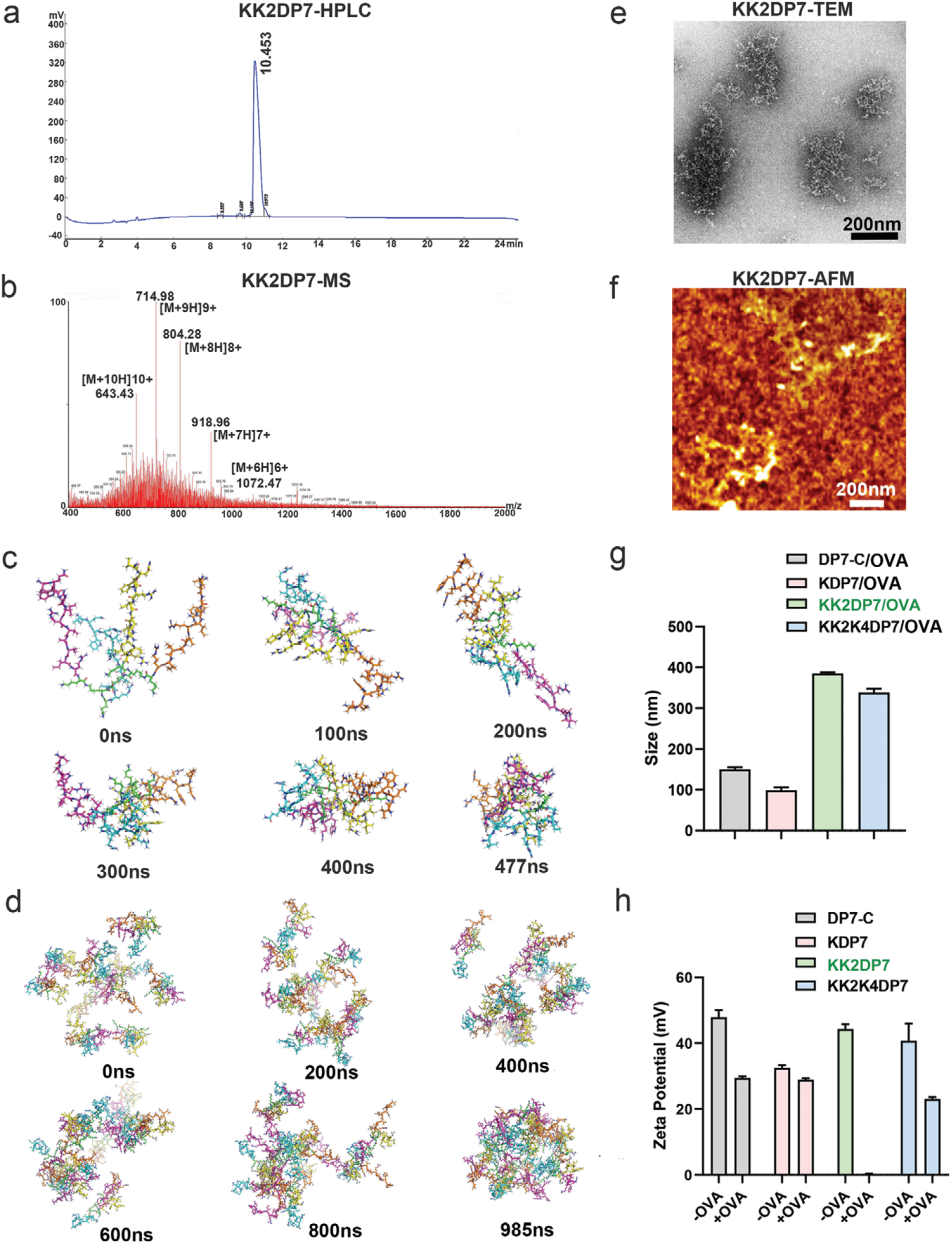
Characterization of KK2DP7. a) High Performance Liquid Chromatography (HPLC) of KK2DP7. b) Mass Spectrometry (MS) of KK2DP7. The structures of the c) monomer model of KK2DP7 and d) multiple peptide model of KK2DP7 at different simulation times. All residues are shown as sticks. The four branches are colored cyan, magenta, yellow, and orange. The Lys residues responsible for connecting the four branches is colored green. The four branches of the monomer model started aggregating after 120 ns and were completely aggregated after 350 ns, and the ten monomers started aggregating after 750 ns and were completely aggregated after 900 ns. e) TEM image of KK2DP7 (60 µg mL^−1^). f) Atomic force microscopy (AFM) image of KK2DP7 (60 µg mL^−1^). g,h) Diameter and zeta potential of DP7 series (60 µg mL^−1^) and DP7 series (60 µg mL^−1^)/OVA (20 µg mL^−1^) complex (*n* = 3). All the values in the present study are presented as the mean ± s.d. unless otherwise indicated in the figure captions.

The synthesis of four‐branched peptides is now a well‐established technique that can be easily quality‐controlled and produced on a large scale. First, we demonstrated the successful synthesis of KK2DP7 by HPLC and MS (**Figure** **2**a,b). Then computer simulation of KK2DP7 was performed to construct a 3D structural model of a single KK2DP7 molecule, and it was found to form a stable dendritic structure in aqueous solution after 477 ns (Figure 2c). Further molecular dynamics simulations were performed on multiple KK2DP7 molecules, and they were found to gradually aggregate into branched polymers in aqueous solution within 985 ns (Figure 2d). The results of transmission electron microscopy (TEM) (Figure 2e) and atomic force microscopy (Figure 2f) also showed that KK2DP7 has a branched polymer structure, which is basically consistent with the molecular dynamics simulation results. KDP7 and KK2K4DP7 were also found to be branched polymers under TEM, and no significant cytotoxicity was observed after KK2DP7 treatment of cells (Figure S2a–c, Supporting Information). Since the DP7 series is positively charged and OVA is negatively charged, the DP7 series and OVA form a complex by electrostatic interaction when incubated with each other. Our experimental results showed that the particle size of all DP7 series combined with OVA was greater than 100 nm (Figure 2g), and the potentials of different ratios of KK2DP7 incubated with OVA were close to neutral at 60:20, indicating that the efficiency of KK2DP7 loaded with OVA is close to 100% at this ratio (Figure 2h, Figure S1d, Supporting Information).

To preliminarily verify whether the particle and zeta potential are the key factors affecting the targeting of LNs, we incubated KK2DP7/OVA at ratios of 40:20, 60:20, and 80:20 and found that the particle sizes were all larger than 100 nm, and the zeta potentials of the complexes incubated at 40:20 and 80:20 ratios were both greater than 20 mV (Figure S1c,d, Supporting Information). After combining the LN targeting data for the three ratios with the particle size and zeta potential data, we preliminarily concluded that the changes in particle size and zeta potential of KK2DP7/OVA do not seem to be the key factors affecting LN targeting.

### 2.2 Endocytosis and Endosome Escape of KK2DP7/OVA in BMDCs

**Figure 3:**
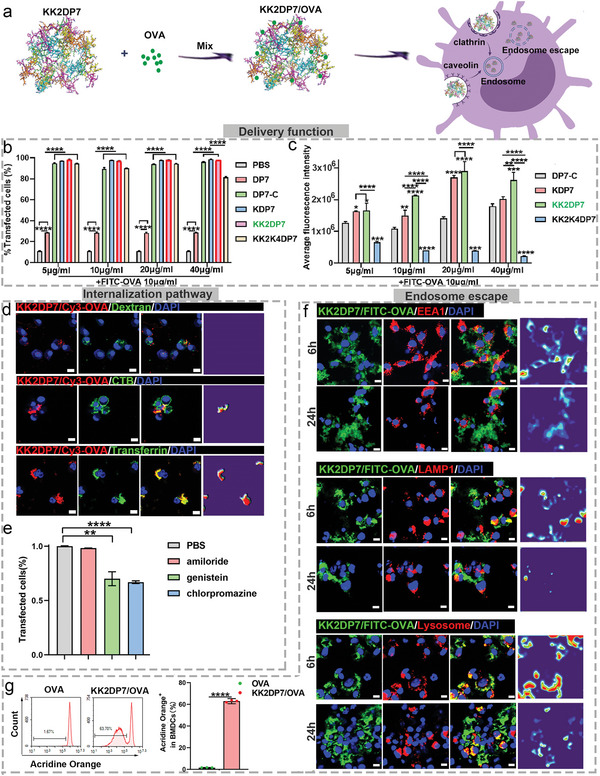
Intracellular behavior of KK2DP7/OVA. a) Schematic diagram describing the intracellular behavior of KK2DP7. b) BMDCs (1 × 10^5^ cells well^−1^ mL^−1^) were incubated with free FITC‐OVA (10 µg) or DP7 series (DP7, DP7‐C, KDP7, KK2DP7, and KK2K4DP7) (5, 10, 20, and 40 µg)/FITC‐OVA (10 µg) for 24 h, and FITC^+^ cells were assessed by flow cytometry analysis. c) The average fluorescence intensity of BMDCs after uptake of DP7 series/FITC‐OVA of b for 24 h. d) Colocalization of three uptake pathway markers with KK2DP7/OVA (dextran, macropinocytosis pathway colocalization probe; CTB, caveolin pathway colocalization probe; transferrin, clathrin pathway colocalization probe). KK2DP7 (20 µg mL^−1^)/Cy3‐OVA (10 µg mL^−1^) was added to BMDCs for 4 h. Then, the dextran (20 mg mL^−1^), CTB (10 µg mL^−1^) and transferrin (50 µg mL^−1^) were used for uptake pathway staining. e) Study of the cellular uptake mechanism of KK2DP7/OVA in the presence of various uptake pathway inhibitors (amiloride, macropinocytosis pathway inhibitor; genistein, caveolin‐mediated endocytosis pathway inhibitor; chlorpromazine, clathrin‐mediated endocytosis pathway inhibitor). BMDCs were pretreated with amiloride (800 µm), chlorpromazine (10 µm), and genistein (200 µm) for 1 h. Then, the KK2DP7 (20 µg mL^−1^)/Cy3‐OVA (10 µg mL^−1^) complex was added for 24 h, and the antigen‐loading efficiency in each group was then determined. f) Colocalization of KK2DP7/OVA with intracellular endosomes. KK2DP7 (20 µg)/FITC‐OVA (10 µg) was added to the BMDCs for 6 and 24 h. For endosomal staining, an antibody specific for the early endosome marker EEA1 was added to the sample for 2 h. For late endosome staining, a PE‐labeled anti‐LAMP1 antibody was added to the sample for 40 min. For lysosomal staining, BMDCs were subjected to LysoTracker Red staining at 37 °C for 2 h. Finally, all samples were stained with DAPI for confocal analysis. Scale bar, 10 µm. g) Flow cytometry analysis of BMDCs stained with acridine orange for 4 h after 24 h of stimulation with KK2DP7/OVA (*n* = 3). The loss of lysosomal staining with acridine orange indicates disruption of cellular lysosomes. All the values in the present study are presented as the mean ± s.d. unless otherwise indicated in the figure captions. One‐way analysis of variance was used for multiple comparisons when more than two groups were compared, and Student's *t*‐test was used for two‐group comparisons. *p < 0.05, **p < 0.01, ***p < 0.001, ****p < 0.0001.

Antigen internalization by DCs is the first step in their subsequent effects. DP7‐C, KDP7, KK2DP7, and KK2K4DP7 increased the internalization efficiency of OVA in BMDCs to more than 90%, and the fluorescence intensity of the KK2DP7/OVA group was significantly higher than that of the other groups (**Figure** **3**a–c). Further exploration of the mechanism of cell internalization revealed that KK2DP7/OVA colocalized with the caveolin‐ and clathrin‐dependent pathway probes CTB and transferrin, and the efficiency of BMDC internalization of KK2DP7/OVA decreased significantly after treatment with the caveolin‐ and clathrin‐dependent pathway inhibitors genistein and chlorpromazine (Figure 3d,e). The complex was not colocalized with the macropinocytosis‐dependent pathway probe dextran, and the antigen‐loading efficiency was not significantly reduced under treatment with the macropinocytosis‐dependent pathway inhibitor amiloride, indicating that the internalization of KK2DP7/OVA by BMDCs occurred via caveolin‐ and clathrin‐dependent pathways (Figure 3a). OVA internalized by DCs must escape endosomes and lysosomes to perform its subsequent function. Therefore, we detected the colocalization of the KK2DP7/OVA complex with endosomes and lysosomes. Colocalization of the complex with endosomes and lysosomes occurred 6 h after antigen loading, and the intensity of colocalization decreased significantly after 24 h (Figure 3f). In addition, we found that the lysosomal escape efficiency of KK2DP7/OVA was greater than 60% by acridine orange staining (Figure 3g).

### 2.3 Activation and Cross‐Presentation of KK2DP7/OVA in BMDCs In Vitro

**Figure 4:**
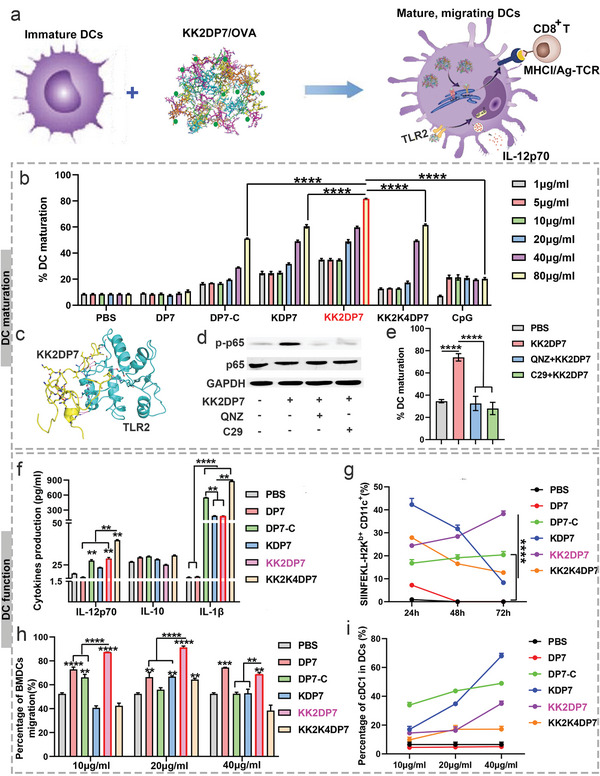
Evaluation of the efficiency of KK2DP7 in promoting BMDC maturation and cytokine secretion, enhancing BMDC antigen presentation, promoting BMDC migration, and increasing the proportion of cDC1^+^ BMDCs. a) Schematic diagram of the mechanism by which KK2DP7 promotes BMDC function. b) The percentages of mature BMDCs after different treatments. BMDCs were treated with DP7, DP7‐C, KDP7, KK2DP7, KK2K4DP7, and CpG (1, 5, 10, 20, 40, and 80 µg mL^−1^) for 24 h, followed by staining with anti‐mouse‐CD11c (HL3), anti‐mouse‐CD86 (GL1) and anti‐mouse‐CD80 (16‐10A1) for 40 min and detection by flow cytometry. c) The 3D binding mode of KK2DP7 and TLR2. KK2DP7 is colored yellow, the surrounding residues in the binding pockets are colored cyan, and the backbone of the receptor is depicted as a cyan cartoon. d) The TLR2‐NF‐*κ*B signaling pathway was activated in KK2DP7‐stimulated BMDCs. Total protein was extracted from BMDCs treated with PBS, KK2DP7 (80 µg mL^−1^), C29 (150 mµ for 2 h) + KK2DP7 (80 µg mL^−1^), or QNZ (10 nm for 2 h) + KK2DP7 (80 µg mL^−1^) for 24 h. The protein lysates (30 µg) were subjected to sodium dodecyl sulfate‒polyacrylamide gel electrophoresis. e) BMDCs were pretreated with the TLR2 inhibitor C29 (150 mµ, MCE, US) and the NF‐*κ*B inhibitor QNZ (10 nm, Selleckchem, US) for 2 h, and then 80 µg mL^−1^ KK2DP7 was added for another 22 h. Flow cytometry experiments revealed that pretreatment of cells with a TLR2 inhibitor (C29) and an NF‐*κ*B inhibitor (QNZ, Targetmol) in vitro inhibited KK2DP7‐mediated cell maturation. f) The supernatants of BMDCs treated with PBS, DP7 (60 µg mL^−1^), DP7‐C (60 µg mL^−1^), KDP7 (60 µg mL^−1^), KK2DP7 (60 µg mL^−1^), or KK2K4DP7 (60 µg mL^−1^) were collected to detect IL‐1*β*, IL‐12p70, and IL‐10 by ELISA. g) The antigen presentation efficiency of BMDCs incubated with DP7 series (20 µg)/OVA (10 µg) for 24–72 h was detected by staining with the monoclonal antibody 25‐D1.16, which recognizes the OVA_257–264_‐H‐2K^b^ complex. h) Transwell assays to detect the percentage of BMDC migration after different treatments. BMDCs treated with 10, 20, or 40 µg mL^−1^ DP7, DP7‐C, KDP7, KK2DP7, or KK2K4DP7 for 24 h were transferred to a Transwell plate. Migration efficiency = total number of lower chamber cells/(total number of lower chamber cells + total number of upper chamber cells). i) The percentage of cDC1s in BMDCs incubated with the DP7 series for 24 h was detected by flow cytometry. All the values in the present study are presented as the mean ± s.d. unless otherwise indicated in the figure captions. One‐way analysis of variance was used for multiple comparisons when more than two groups were compared, and Student's *t*‐test was used for two‐group comparisons. **p < 0.01, ***p < 0.001, ****p < 0.0001.

DCs are the most powerful APCs, and vaccines targeting LNs must be internalized by DCs and stimulate DCs to mature before they can be effectively presented to T cells by DCs. In addition, cytokines secreted by DCs, especially IL‐12p70 and IL‐1*β*, are closely related to vaccine efficacy and promote CTL differentiation and CD8^+^ T‐cell proliferation.^[^ ^6^ ^]^ The results of the previous experiments showed that OVA itself did not affect DC maturation, cytokine secretion, DC migration or DC classification. Therefore, in addition to the antigen presentation experiments, we used KK2DP7 to replace KK2DP7/OVA. Compared with the other control groups, the KK2DP7 group showed a significantly increased proportion of mature BMDCs, and the ability to promote BMDC maturation was not weaker than that of the commonly used adjuvant CpG (TLR9 agonist) group (**Figure** **4**b). In a previous study, we demonstrated that DP7‐C promotes BMDC maturation through activation of the TLR2‐NF‐*κ*B signaling pathway.^[^ ^4b^ ^]^ We therefore speculate that this pathway may also be responsible for the promotion of BMDC maturation by KK2DP7. Thus, to further confirm that KK2DP7 exerts its effects by acting on TLR2, we further investigated it by molecular docking (Figure 4c). The final stable KK2DP7/TLR2 complex is shown in Figure 3d. The binding free energy of the KK2DP7/TLR2 interaction is −80.55 ± 1.22 kcal mol^−1^ in aqueous environments (Table S1, Supporting Information). Furthermore, we confirmed that KK2DP7‐induced activation of NF‐*κ*B and BMDC maturation were significantly inhibited after pretreatment with QNZ (an NF‐*κ*B inhibitor) and C29 (a TLR2 inhibitor), suggesting that KK2DP7 is also responsible for activating BMDCs via interaction with TLR2 to activate downstream NF‐*κ*B signaling pathways (Figure 4a,d,e). In addition, the KK2DP7 group also showed obvious advantages compared with the untreated group in stimulating BMDC secretion of IL‐12p70 and IL‐1*β* (Figure 4f). Furthermore, the antigen presentation efficiency of the KK2DP7/OVA group increased with time. After 72 h, the antigen presentation efficiency of the KK2DP7/OVA group was obviously superior to that of the other groups (Figure 4g). The migration of antigen‐containing DCs to the LNs as well as migration within the LNs has been shown to be positively correlated with vaccine efficacy.^[^ ^7^ ^]^ The DP7 series had a clear ability to promote DC migration when administered at different concentrations. In addition, KK2DP7 maintained the ability to promote BMDC migration at low, medium and high doses and was significantly better than the other treatments (Figure 4h). Furthermore, to induce an effective antitumor cytotoxic T lymphocyte (CTL) immune response, the nanovaccine should be delivered to cDC1‐type DCs.^[^ ^8^ ^]^ The results showed that the DP7 series showed a unique advantage in inducing the differentiation of DCs to the cDC1 type (Figure 4i).

### 2.4 Activation and Cross‐Presentation of KK2DP7/OVA in DCs in LNs

**Figure 5:**
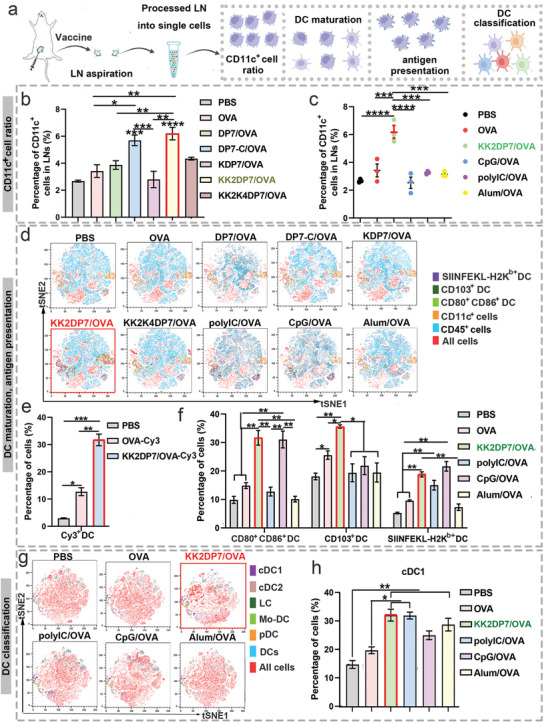
LN targeting mechanism of KK2DP7/OVA and evaluation of DC function in LNs. a) Schematic diagram of the experimental design. b,c) Proportion of CD11c^+^ cells in the LNs at 24 h after subcutaneous immunization with DP7 series (60 µg per mouse)/OVA, CpG (40 µg per mouse)/OVA, poly(I:C) (50 µg per mouse)/OVA, or alum (100 µg per mouse)/OVA. (OVA = 20 µg per mouse). d) TSNE analysis of DCs (including CD80^+^CD86^+^ DCs, CD103^+^ DCs, and SIINFEKL‐H2Kb^+^ DCs) in the LNs 24 h after immunization with different regimens. e) OVA‐Cy3‐positive DCs in the LNs 24 h after subcutaneous immunization with KK2DP7 (60 µg per mouse)/OVA (20 µg per mouse). f) Percentage of CD80^+^CD86^+^ DCs, CD103^+^ DCs, and SIINFEKL‐H2Kb^+^ DCs in the LNs 24 h after immunization with different regimens. g,h) Representative flow cytometry data and statistical data for DC classification and cDC1^+^ DCs in the LNs 24 h after immunization with different regimens. All the values in the present study are presented as the mean ± s.d. unless otherwise indicated in the figure captions. One‐way analysis of variance was used for multiple comparisons when more than two groups were compared, and Student's *t‐*test was used for two‐group comparisons. *p < 0.05, **p < 0.01, ***p < 0.001, ****p < 0.0001.

In the in vivo study, we further validated the mechanism and efficiency of KK2DP7/OVA LN targeting by DC internalization, stimulation of DC maturation, antigen presentation, and induction of cDC1‐type DCs (**Figure** **5**a). First, combined with the previous particle size and zeta potential results, we preliminarily hypothesized that KK2DP7/OVA might enter LNs via transport by immune cells. Therefore, we compared the proportions of DCs in LNs after subcutaneous injection of the complex and other controls and found that the proportion of DCs in LNs, the efficiency of DC uptake of OVA in LNs and the proportion of migrating CD103^+^ DCs in LNs in the KK2DP7/OVA group were all significantly increased (Figure 5b–f, Figure S3, Supporting Information). We further analyzed DC function and DC typing in the LNs and found that the KK2DP7/OVA immunization group did not show a weaker effect than the poly (I:C)/OVA, CpG/OVA and alum/OVA groups in terms of DC maturation and antigen presentation (Figure 5d–f). In terms of DC typing, only cDC1s were significantly upregulated after KK2DP7/OVA immunization, and the proportion of cDC1s after KK2DP7/OVA immunization was not lower than that after immunization with the poly (I:C), CpG and alum adjuvants (Figure 5g,h, Figure S4a,b, Supporting Information). In terms of macrophages, the KK2DP7/OVA group did not show significant changes (Figure S5a–c, Supporting Information). These results suggest that the targeting of LNs by KK2DP7/OVA is achieved via the transport of DCs, and the complex subsequently functions by stimulating DC maturation, increasing the proportion of cDC1‐type DCs and improving the efficiency of DC antigen presentation.

### 2.5 Improved Antitumor Response with the KK2DP7/OVA‐Based Nanovaccine

**Figure 6:**
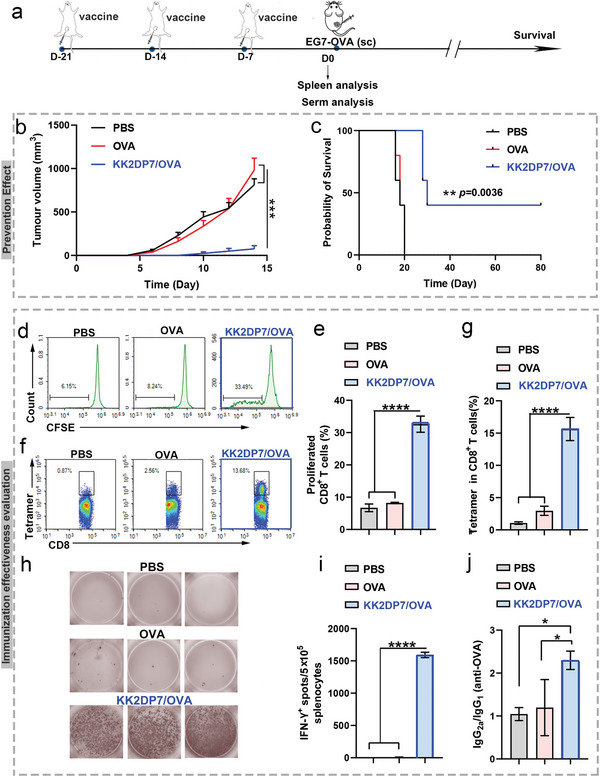
Evaluation of the immune efficacy of the KK2DP7/OVA nanovaccine and observation of tumor growth inhibition and survival in tumor‐bearing mice in a preventive model. a) Schematic of the tumor challenge experimental design. b) Average tumor growth curves for EG7‐OVA tumors in mice after various treatments, as indicated. c) Morbidity‐free survival of different groups of mice with EG7‐OVA tumors after various treatments. d,e) Representative flow cytometry plots and statistical data showing proliferating (carboxyfluorescein succinimidyl ester (CFSE) dilution) CD8^+^ T cells in the spleen after SIINFEKL (10 µg mL^−1^) stimulation in mice sacrificed on day 7 post immunization. f,g) Representative flow dot plots and statistical data for H‐2K^b^/SIINFEKL tetramer staining of CD8^+^ T cells in the spleen on day 7 post immunization. h,i) IFN‐*γ* spot‐forming cells and statistical data from restimulated splenocytes determined by the ELISPOT assay on day 7 post immunization. j) Ratios of OVA‐specific IgG2a:IgG1 in the sera of immunized mice on day 7 post immunization. All the values in the present study are presented as the mean ± s.d. unless otherwise indicated in the figure captions. One‐way analysis of variance was used for multiple comparisons when more than two groups were compared, and Student's *t*‐test was used for two‐group comparisons. Survival curves were obtained using the Kaplan–Meier method and compared by the log‐rank test. *p < 0.05, ***p < 0.001, ****p < 0.0001.

To further evaluate the immunization efficacy and the immunoprotective effect of the KK2DP7/OVA nanovaccine, the spleen and serum of some immunized mice were collected for antigen‐specific immune response detection, and other immunized mice were challenged with OVA‐expressing EG7‐OVA tumor cells 7 days after the final immunization (Figure 5a). The results showed that in mice injected with KK2DP7/OVA, the average tumor volume was considerably smaller than that in the other mice at all‐time points, and survival was significantly prolonged (**Figure** **6**b,c, Figure S6a–c, Supporting Information). The KK2DP7/OVA nanovaccine could induce effective antigen‐specific CD8^+^ T‐cell proliferation (Figure 6d,e). Notably, the frequency of SIINFEKL‐MHC‐I tetramer^+^ CD8^+^ T cells exhibited a fivefold increase in the KK2DP7/OVA group compared with the OVA group (Figure 6f,g). Typically, stronger antigen‐specific T‐cell‐mediated immune responses occur upon repeated exposure to the same antigen.^[^ ^9^ ^]^ During this process, T cells rapidly eliminate any cells that express the specific surface antigen. Upon repeated exposure to the same antigen, OVA peptide (SIINFEKL) and IFN‐*γ* ELISPOT (enzyme‐linked immune absorbent spot) responses in splenocytes from mice immunized with KK2DP7/OVA were found to be significantly increased, which demonstrates the robust in vivo antigen‐specific T‐cell immune responses triggered by the KK2DP7/OVA nanovaccine (Figure 6h,i). Furthermore, KK2DP7/OVA elicited Th1‐polarized immune responses, as shown by an OVA‐specific immunoglobulin IgG_2a_/IgG_1_ ratio of >1 (Figure 6j).

### 2.6 The KK2DP7/OVA and KK2DP7/Neoantigen Nanovaccines Inhibit Tumor Growth and Prolong Survival in Tumor‐Bearing Mice

**Figure 7:**
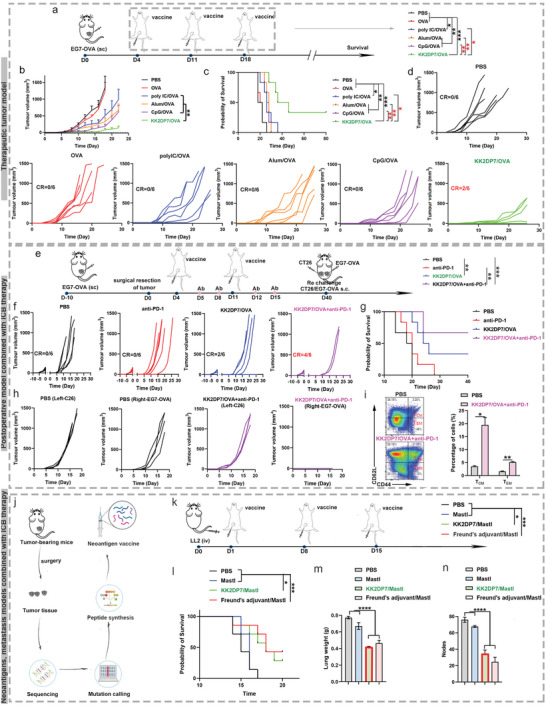
The KK2DP7/OVA and KK2DP7/neoantigen nanovaccines inhibit tumor growth and prolong survival in tumor‐bearing mice. a) Schematic of the tumor challenge experimental design in tumor‐bearing mice. b) Average tumor growth curves for EG7‐OVA tumors in mice after various treatments, as indicated. c) Morbidity‐free survival of different groups of mice with EG7‐OVA tumors after various treatments. d) Individual tumor growth curves from b). e) Schematic of the tumor challenge experimental design in synergy of the KK2DP7/OVA nanovaccine with ICB therapy. f) Individual tumor growth curves for EG7‐OVA tumors in mice after various treatments, as indicated. g) Morbidity‐free survival of different groups of mice with EG7‐OVA tumors after various treatments. h) On day 40, the mice that survived were subcutaneously injected with 1 × 10^6^ EG7‐OVA and CT26 cells to evaluate immunological memory. Growth of tumors in the right flank of EG7‐OVA and left flank of CT26. i) Representative flow dot plots and statistical data of T_EM_ and T_CM_ in the spleen analyzed by flow cytometry (gated on CD3^+^ CD8^+^ T cells) on day 60. j) Schematic diagram of neoantigen screening. k) Treatment scheme. l) Survival of different groups of mice with LL2 tumors after various treatments. m) Lung weight of each group. n) Statistics on the number of nodules on the lung surface. All the values in the present study are presented as the mean ± s.d. unless otherwise indicated in the figure captions. One‐way analysis of variance was used for multiple comparisons when more than two groups were compared, and Student's *t*‐test was used for two‐group comparisons. SC: subcutaneous. CR was defined as complete tumor disappearance. Survival curves were obtained using the Kaplan–Meier method and compared by the log‐rank test. *p < 0.05, **p < 0.01, ***p < 0.001, ****p < 0.0001.

The therapeutic effect of the KK2DP7/OVA nanovaccines was also evaluated using the EG7‐OVA tumor model (**Figure** **7**a). Poly (I:C), a TLR‐3 agonist; CpG oligonucleotide, a TLR‐9 agonist; and aluminum (alum) were chosen as the control adjuvants. In the PBS and OVA control groups, all the animals died within 30 days. The poly (I:C)/OVA, CpG/OVA and alum/OVA groups exhibited a small immunotherapeutic effect. In contrast, KK2DP7/OVA‐immunized mice maintained an evidently high survival rate of 33.3% up to 60 days (Figure 7b–d). The body weights of the mice were not impacted by the treatment (Figure S7a,b, Supporting Information). In addition, H&E staining of major organs collected from mice 7 days after three subcutaneous injections of KK2DP7/OVA was performed. No noticeable signs of organ damage appeared in any of the major organs of the mice (Figure S8, Supporting Information).

Then, we propose a general strategy of cancer vaccination combined with ICB therapy for postsurgical immunotherapy of recurrent tumors. Therefore, a recurrent EG7‐OVA tumor model was established by inoculating EG7‐OVA tumor cells subcutaneously into each mouse and then surgically removing the tumor on day 0 (Figure 7e). Compared with untreated mice, mice vaccinated with KK2DP7/OVA showed partially delayed tumor recurrence (Figure 7f). Of the 6 mice, 33.33% became tumor free and survived up to 40 days (Figure 7g). Moreover, although anti‐PD‐1 treatment alone appeared to be less effective, KK2DP7/OVA vaccination combined with anti‐PD‐1 showed impressive therapeutic effects in inhibiting tumor recurrence. The body weights of the mice were not impacted by the treatment (Figure S7c, Supporting Information). The tumor‐free survival rate in this group also showed a dramatic increase to 66.67% within 40 days (Figure 7g), which demonstrates the great promise of combining KK2DP7‐based cancer vaccines with ICB therapy to treat postsurgical tumor recurrence, which is a major challenge in clinical cancer treatment. To assess the immune memory effects, mice that survived on day 40 in the KK2DP7/OVA+anti‐PD‐1 group were rechallenged with CT26 and EG7‐OVA cells. Compared with the untreated mice, the tumor growth on the mice that survived after combination therapy showed no reoccurrence of the secondary EG7‐OVA tumor but showed no difference in CT26 tumor (Figure 7h). To further verify the immunological memory responses induced by our vaccination‐based combination therapy, in the KK2DP7/OVA+anti‐PD‐1 group, spleen cells were collected on day 60 to assess memory CD8^+^ T cells. Intriguingly, for the tumor‐free mice that survived to 60 days after EG7‐OVA cell challenge (KK2DP7/OVA+anti‐PD‐1), the percentage of effector memory T cells (*T*_EM_) and central memory T cells (*T*_CM_) in the CD3^+^CD8^+^ T cells showed a significant increase compared with that in the control mice (Figure 7i).

Furthermore, our proposed general strategy of combining cancer vaccines with ICB therapy has shown strong potential for application in the treatment of neoantigen‐based lung metastasis models. Here, we selected *Mastl*_D366Y (MUT/WT sequence: LSPIH(Y/D)SSA), a neoantigen derived from LL2, which was screened and verified by sequencing technology and bioinformatics in previous experiments,^[^ ^10^ ^]^ in combination with KK2DP7 or Freund's adjuvant for immunization (Figure 7j,k). The results showed that the mice in the combined treatment group had significantly longer survival, lower lung weight and lower number of lung nodules (Figure 7l–n, Figure S7d, Supporting Information). The therapeutic impact of KK2DP7 was comparable to that of Freund's adjuvant.

## 3 Discussion

This study focuses on the frontier hotspots and urgent needs of tumor immunotherapy. Although personalized tumor vaccines have made significant breakthroughs in clinical trials of some malignant tumors, the clinical efficacy of the vaccines still needs to be improved.^[^ ^11^ ^]^ Therefore, the key to improving the clinical efficacy of personalized tumor vaccines is to effectively enhance the immune effect of the vaccines. In addition to the accuracy of neoantigen prediction, the development and selection of adjuvants and delivery systems in personalized tumor vaccines are also of paramount importance influencing the immune effect of vaccines.^[^ ^12^ ^]^ First, the development of safe and effective novel tumor vaccine adjuvants is essential to enhance the immune effect of neoantigen vaccines.^[^ ^12a^ ^]^ In addition, the low targeted delivery efficiency of the vaccine delivery system to lymphoid organs is the “culprit” that limits the immune efficacy of vaccines and the rapid induction of antitumor immune responses.^[^ ^2^, ^12^, ^13^ ^]^ Therefore, the development of innovative safe and effective vaccine adjuvants and lymph node‐targeted delivery systems is imperative to improve the clinical efficacy of neoantigen vaccines. To address the abovementioned bottlenecks, the present study has taken an alternative approach to the design of vaccine adjuvants and lymph node‐specific targeted delivery systems. Based on the modification of the previously developed peptide with the dual functions of delivery vehicle and immune adjuvant, we designed a dendritic peptide (KK2DP7) with a simple preparation method and integrated immune adjuvant and lymph node targeting delivery functions to enhance the immune and antitumor effects of vaccines. Our design has the following advantages. First, as an immune adjuvant, KK2DP7 can activate both humoral and cellular immune responses to assist in the activation of antigen‐specific immune responses. Second, as a lymph node‐targeted delivery carrier, KK2DP7 can efficiently target the delivery of antigens to lymph nodes and rapidly induce antitumor immune responses. In addition, we combined the KK2DP7/neoantigen vaccine with immune checkpoint inhibitor therapy to rapidly induce systemic antitumor immune responses while relieving the body of immune suppression, and the “multi‐linkage” combination improved the immune effect and clinical efficacy of the neoantigen vaccine.

Specifically, in this experiment, we designed a series of branched DP7 molecules based on previous research on DP7 and DP7‐C and explored the differences in the effect of combination with OVA as a tumor vaccine for LN targeting. We found that KK2DP7 significantly enhanced the LN‐targeting effect of OVA, while other DP7s (DP7, DP7‐C, KDP7, KK2K4DP7) did not have similar effects. Therefore, we attempted to elucidate the mechanism of LN targeting of KK2DP7 through a series of experiments. First, LN targeting is reported to be related to the particle size of the nanodelivery system.^[^ ^1^, ^14^ ^]^ Generally, complexes less than 10 nm are easily cleared by blood and cannot enter LNs. Complexes of 10–100 nm are considered to enter LNs through lymphatic vessels, while complexes greater than 100 nm are considered to enter LNs through transport by immune cells.^[^ ^1^, ^14^ ^]^ Our experimental results showed that the particle size of all DP7 series combined with OVA was greater than 100 nm, so if they entered the LNs, it was likely via transport by immune cells. Second, the charge of nanoparticles is also related to the LN targeting effect. It is generally believed that negatively charged and neutrally charged nanoparticles are more likely to enter LNs because they are not affected by negatively charged glycosaminoglycans in the interstitial space.^[^ ^15^ ^]^ The results showed that the potentials of different ratios of KK2DP7 incubated with OVA were close to neutrality at 60:20, but there was no significant difference in the LN targeting effect among the three ratios. This finding suggests that the charge of the KK2DP7/OVA complex is not the key factor affecting LN targeting. This may be related to the fact that the complex may be mainly transported into the LNs by immune cells.

To verify whether the KK2DP7/OVA complex is transported into the LNs by immune cells, we analyzed the proportions of DCs and macrophages in the LNs of mice injected with the KK2DP7/OVA complex and other controls. The proportion of macrophages in the LNs of mice in the KK2DP7/OVA complex group did not change significantly, while the proportion of DCs was significantly increased. It was preliminarily demonstrated that KK2DP7/OVA is transported to the LNs by DCs. Notably, the proportion of DCs in the LNs after subcutaneous injection in the DP7‐C group was also significantly increased; however, no obvious LN targeting was observed in this group. Furthermore, considering the analysis of DC function in the LNs, we believe that the reason may be that KK2DP7/OVA is transported into the LNs by DCs, while DCs ingesting the complex in the DP7‐C/OVA group may remain at the injection site after injection, and the inflammatory cytokines released by them induce other DCs, which do not ingest DP7‐C/OVA, to be recruited into the LNs. DCs in the LNs of the KK2DP7/OVA group were obviously stimulated to mature and present antigens, while DCs in the DP7‐C/OVA group were mostly immature DCs that did not present antigens. In addition, the in vitro results showed that under the condition of a high concentration of DP7‐C, the DC migration efficiency was not significantly different from that of the control group, while KK2DP7 maintained the ability to promote DC migration at all concentrations. Therefore, the obvious difference in LN targeting between the KK2DP7 group and the DP7‐C group when the proportion of DCs in the LNs was increased may have been due to the difference in the transport and recruitment of immune cells. Overall, based on our experimental results, we tentatively believe that the lymph node targeting advantage of KK2DP7 over other DP7 series may lie in its facilitation of more efficient migration of DCs that take up antigen to the lymph nodes. However, we acknowledge that we cannot rule out the possibility of in vivo deformation of nanoparticles or deeper causes, and we currently do not have a better way to explore these possible causes. We will conduct more complex and in‐depth studies on this aspect in subsequent experiments, with the aim of providing new ideas for the design of lymph node‐targeting nanoparticles.

In conclusion, in this study, we developed a simple dendrimer KK2DP7 polymer nanovaccine to induce robust antitumor immunity. The results showed that KK2DP7 could significantly enhance the accumulation of OVA in LNs through the trafficking of DCs. As a delivery vehicle, it can efficiently deliver OVA to DCs via caveolin‐ and clathrin‐dependent pathways. As an immune adjuvant, it can stimulate DC maturation by activating the TLR2‐NF‐*κ*B signaling pathway. Therefore, the KK2DP7/antigen system can induce a robust tumor‐specific immune response in vivo. In animal experiments, the antitumor effect of KK2DP7/OVA was superior to that of commonly used adjuvants such as poly(I:C), CpG, and aluminum adjuvant combined with OVA. This may be related to the ability of KK2DP7 to deliver antigen into dendritic cells more efficiently than these adjuvants. Strikingly, KK2DP7/OVA combined with anti‐PD‐1 antibody showed excellent antitumor effects and was able to prevent tumor recurrence in a postoperative recurrent tumor model. In addition, KK2DP7/neoantigen combined with anti‐PD‐1 antibody also showed excellent antitumor effects in an LL2 venous metastasis model. These results strongly demonstrate the advantages of KK2DP7 as a nanovaccine for cancer treatment. This enhanced personalized anticancer vaccine may have important clinical translational value in the context of personalized postoperative cancer immunotherapy. In addition to the protein‐based vaccines reported in this study, dendrimer KK2DP7 polymers may be useful in the development of peptide vaccines, DC vaccines, and nucleic acid vaccines (e.g., mRNA vaccines), for which transmembrane delivery, DC activation, and antigen presentation are also necessary processes for vaccine efficacy. Furthermore, in addition to anticancer vaccines, the application of the dendrimer KK2DP7 polymer may be further expanded to generate other types of important vaccines, such as vaccines against viral infections such as COVID‐19.

## 4 Experimental Section

### Cells and Animals

Roswell Park Memorial Institute (RPMI) 1640 medium containing 100 units mL^−1^ streptomycin and penicillin (PS) and 10% fetal bovine serum (FBS) was used to culture bone marrow‐derived DCs (BMDCs) and EG7‐OVA cells (American Type Culture Collection, Manassas, VA, USA). All cells were cultured in a cell incubator containing 5% CO_2_ at 37 °C. RPMI 1640 medium FBS and PS were all purchased from Thermo Fisher Scientific. Female 6‐ to 8‐week‐old C57BL/6J mice were purchased from HFK Bioscience (Beijing, China). All animal procedures were approved and controlled by the Institutional Animal Care and Treatment Committee of Sichuan University and conducted according to the Animal Care and Use Guidelines of Sichuan University.

BMDCs were obtained from 4‐ to 6‐week‐old C57BL/6J female mice according to a previously reported protocol.^[^ ^16^ ^]^ Briefly, after treating bone marrow cells with red blood cell lysis buffer, fresh RPMI 1640 complete medium containing 20 ng mL^−1^ granulocyte‐macrophage colony‐stimulating factor (GM‐CSF; PrimeGene Biotechnology, Shanghai, China) was added to 3 × 10^6^ mouse bone marrow cells. On day 8, BMDCs were collected for further use.

### Preparation and Characterization of the Peptide and Peptide/OVA Complex

DP7 (VQWRIRVAVIRK), DP7‐C (cholesterol‐DP7), KDP7 (VQWRIRVAVIRKK), KK2DP7 ((VQWRIRVAVIRK)_2_KK) and KK2K4DP7 (((VQWRIRVAVIRK)_2_K)_2_KK) were synthesized, purified (>90%) and verified by Apeptide Co., Ltd. (Shanghai, China) (Figure S1a–c, Supporting Information). The HPLC and MS results were obtained from Apeptide Co., Ltd. (Shanghai, China). The structural formula of KK2DP7 was provided by Wecomput Technology Co., Ltd. (Beijing, China) for computer simulation of its state in aqueous solution for preliminary characterization. The abovementioned polypeptide and OVA were dissolved in deionized water, and the complex could be formed by simple mixing. The diameter and zeta potential were measured by a Zetasizer Nano ZS (Malvern Panalytical Co. Ltd). All results are the means from three experiments. The morphological characteristics of KDP7, KK2DP7, and KK2K4DP7 were examined by using TEM (H6009IV, Hitachi, Tokyo, Japan) and atomic force microscopy (AFM; NSK Ltd., Tokyo, Japan).

### Structural Preparation of Peptides

The peptide monomer with four branches was prepared in the Molecular Operating Environment (MOE2020) package. The 3D structures of the four branches were first constructed separately based on their sequences. Then, seven cross‐linked lysines were generated to connect the four previously built branches. Finally, the constructed peptide monomer was subjected to preliminary optimization under the MMFF94 molecular field. The final optimized peptide monomer model is presented in Figure 2b.

The multiple peptide model was prepared in the Packmol package. The structure of the peptide monomer after ≈500 ns of molecular dynamics simulations (the detailed method for the molecular dynamics simulations is shown below), as shown in Figure 2b, was selected to build the multiple peptide model. By employing the Packmol package, ten selected peptide monomer structures were randomly inserted into a 10 nm × 10 nm × 10 nm cubic box. The 10 randomly distributed peptide monomers that were deemed the multiple peptide model are presented in Figure 2c.

Before the molecular dynamics simulations, the above constructed peptide monomer structure was first neutralized by the addition of neutralizing ions (Na^+^ or Cl^−^) on the peptide surfaces and solvated in a rectangular box with a 10 Å buffer distance between the solvent box wall and the nearest peptide atoms. At the same time, for the multiple peptide model, the water molecules were directly put into the 10 nm × 10 nm × 10 nm cubic box to fill the empty space, and the same neutralizing ions (Na^+^ or Cl^−^) were added to the peptide surfaces. All the above setups were driven by the tleap program of the AmberTools package, and the final obtained solvated peptide monomer model and multiple peptide model were employed to run the molecular dynamics simulations.

### Molecular Dynamics Simulations

All molecular dynamics simulations were performed using AMBER20. The AMBER FF14SB force fields were applied to describe the peptide behaviors, and the TIP3P model was employed for the water molecules. The SHAKE algorithm was used to restrict all covalent bonds involving hydrogen atoms with a time step of 2 fs. The particle mesh Ewald (PME) method was employed to treat long‐range electrostatic interactions with a cutoff value equal to 12 Å. For each solvated model, two steps of minimization were performed before the heating step. The first 4000 cycles of minimization were performed with all heavy atoms restrained at 50 kcal/(mol Å^2^), whereas solvent mole cubes and hydrogen atoms were free to move. Then, nonrestrained minimization was carried out involving 2000 cycles of steepest descent minimization and 2000 cycles of conjugated gradient minimization. Afterward, the whole model was first heated from 0 to 300 K in 100 ps using Langevin dynamics at a constant volume and then equilibrated for 100 ps at a constant pressure of 1 atm. A weak constraint of 10 kcal/(mol Å^2^) was used to restrain all the heavy atoms during the heating steps. In the production phase, ≈500 ns simulations for the peptide monomer model and ≈1 µs simulations for the multiple peptide model were carried out under the NVT ensemble.

### Lymph Node Accumulation of the Peptide/OVA Complex

To detect the accumulation of the peptide/OVA complex in the lymph nodes after subcutaneous injection, Cy7‐OVA (20 µg) and DP7 (60 µg), DP7‐C (60 µg), KDP7 (60 µg), KK2DP7 (40, 60, and 80 µg), KK2K4DP7 (60 µg), and CpG (40 µg) were co‐incubated and then injected subcutaneously near the right of the lymph nodes. The left and right lymph nodes at the injection sites were removed after 4 or 24 h, and the presence of fluorescence in the lymph nodes was detected by ex vivo imaging using a PerkinElmer IVIS Lumina III. The fluorescence intensity of each group was quantified with the instrument's own software.

### Antigen‐Loading Efficiency of OVA and the Cellular Uptake Pathway Assay

BMDCs (5 × 10^5^ cells well^−1^) were seeded in a 24‐well plate 24 h before antigen loading. Next, FITC‐OVA (10 µg mL^−1^) and DP7, DP7‐C, KDP7, KK2DP7, and KK2K4DP7 (5, 10, 20, and 40 µg mL^−1^) incubated in 1640 complete medium were added to the plate for another 24 h. Then, the antigen‐loading efficiency was evaluated by flow cytometry.

To determine the cellular internalization mechanism of the KK2DP7/OVA complex, KK2DP7 (20 µg mL^−1^)/Cy3‐OVA (10 µg mL^−1^) was added to BMDCs for 4 h. Then, the macropinocytosis pathway colocalization probe dextran Texas Red (20 mg mL^−1^, staining at 37 °C for 20 min, Invitrogen), caveolin pathway colocalization probe CT‐B (10 µg mL^−1^, staining at 4 °C for 15 min, Invitrogen) and clathrin pathway colocalization probe transferrin (50 µg mL^−1^, staining at 37 °C for 2 h, Jackson) were used for uptake pathway staining. The stained cells were fixed with 4% paraformaldehyde, sealed with a DAPI‐containing anti‐fluorescence quencher (Solarbio) and imaged via laser confocal microscopy (Leica).

To further investigate the cellular internalization mechanism of the KK2DP7/OVA complex, BMDCs were pretreated with different inhibitors: amiloride (800 µm, an inhibitor of the macropinocytosis pathway), chlorpromazine (10 µm, an inhibitor of the clathrin‐mediated endocytosis pathway), and genistein (200 µm, an inhibitor of the caveolin‐mediated endocytosis pathway) for 1 h. Then, the KK2DP7 (20 µg mL^−1^)/Cy3‐OVA (10 µg mL^−1^) complex was added for 24 h, and the antigen‐loading efficiency of the KK2DP7/OVA complex in each group was determined by flow cytometry.

### Endosomal and Lysosomal Escape Assay

The endosomal and lysosomal escape assays were conducted as previously described.^[^ ^17^ ^]^ Briefly, BMDCs (1 × 10^5^ cells well^−1^) were seeded in a 24‐well plate containing cell climbing films for 24 h. Next, KK2DP7 (20 µg)/FITC‐OVA (10 µg) was added to the cells for 6 and 24 h. For endosomal staining, the cells were fixed with 4% paraformaldehyde for 20 min at room temperature and washed three times with PBS for 5 min each. For early endosome staining, cells were treated with 0.1% Triton X‐100 for 20 min at room temperature and washed three times with PBS for 5 min each. Subsequently, an antibody specific for the early endosome marker EEA1 (Invitrogen, USA) (1:100) was added to the sample for 2 h at room temperature and thoroughly washed five times for 5 min each with PBS. Then, the PE‐labeled secondary antibody (BD, USA) (1:100) was added to the sample for another 40 min at room temperature prior to three washes with PBS for 5 min each. For late endosome staining, a PE‐labeled anti‐LAMP1 antibody (BD, USA) (1:100) was added to the sample for 40 min at 4 °C prior to three washes with PBS for 5 min each. For lysosomal staining, BMDCs in 24‐well plates were subjected to LysoTracker Red (Beyotime Biotechnology) staining at 37 °C for 2 h prior to three washes with PBS for 5 min each. Then, all samples were stained with DAPI (Beyotime Biotechnology, China) for 30 min prior to three washes with PBS for 5 min each. Finally, all the cell climbing films were acquired for confocal analysis.

To detect the disruption of cellular lysosomes, BMDCs were treated with KK2DP7 (20 µg)/OVA (10 µg) for 24 h. Then, flow cytometry analysis of BMDCs stained with acridine orange for 4 h was performed. The loss of lysosomal staining with acridine orange indicated disruption of cellular lysosomes.

### Flow Cytometry Assay of BMDC Function In Vitro

To detect BMDC maturation and cDC1 proportions, BMDCs were treated with DP7, DP7‐C, KDP7, KK2DP7, KK2K4DP7, and CpG (1, 5, 10, 20, 40, and 80 µg mL^−1^) for 24 h, followed by staining with anti‐mouse‐CD11c (HL3), anti‐mouse‐CD86 (GL1), and anti‐mouse‐CD80 (16‐10A1) or anti‐mouse‐CD11c (HL3), anti‐mouse‐CD8 (53‐6.7) antibodies (BD, US) for 40 min and detection by flow cytometry. To detect the efficiency of antigen presentation, BMDCs were incubated with KK2DP7 (20 µg)/OVA (10 µg) for 24–72 h, stained with the anti‐mouse‐CD11c (HL3) antibody and the monoclonal antibody 25‐D1.16 for 40 min, and then detected by flow cytometry.

To verify whether KK2DP7 promotes BMDC maturation through the TLR2 and NF‐*κ*B signaling pathways, BMDCs were pretreated with the TLR2 inhibitor C29 (150 mµ, MCE, US) and the NF‐*κ*B inhibitor QNZ (10 nm, Selleckchem, US) for 2 h, added 80 µg mL^−1^ KK2DP7 to treat BMDCs for 22 h, and used flow cytometry to detect the proportion of mature BMDCs. Flow cytometry was performed on a FACSAria SOPR (BD, USA) flow cytometer, and the data were analyzed with FlowJo 10.8.1 software.

### Cell Migration Assay

In in vitro migration experiments, BMDCs (1 × 10^5^ mL^−1^) treated with 10, 20, or 40 µg mL^−1^ DP7, DP7‐C, KDP7, KK2DP7, or KK2K4DP7 for 24 h were collected and transferred to a Transwell plate (NEST Biotechnology Co. Ltd). The upper chamber contained 1 × 10^5^ cells in 100 µL of RPMI 1640 medium. The lower chamber contained 500 µL of RPMI 1640 medium supplemented with 10% FBS (VivaCell, Shanghai, China) + CCL19 (250 ng mL^−1^) + CCL21 (250 ng mL^−1^). The cells in the upper chamber and lower chamber were counted after 24 h (Countstar). Migration efficiency = total number of lower chamber cells/(total number of lower chamber cells + total number of upper chamber cells).

### ELISA

The supernatants of BMDCs (5 × 10^5^ mL^−1^) treated with PBS, DP7 (60 µg mL^−1^), DP7‐C (60 µg mL^−1^), KDP7 (60 µg mL^−1^), KK2DP7 (60 µg mL^−1^), or KK2K4DP7 (60 µg mL^−1^) were collected, and the levels of IL‐12p70, IL‐10, and IL‐1*β* were detected with an ELISA kit (Zicbio, Shanghai; Beijing Solarbio Science & Technology Co., Ltd) according to the vendor's instructions. For serum IgG determination, sera from mice immunized with KK2DP7 (60 µg)/OVA (20 µg) three times (once a week) were separated for experiments on day 7 post‐immunization. The levels of OVA‐specific IgG1 and IgG2a in the serum were also measured by ELISA following a previously reported protocol.^[^ ^18^ ^]^ All tests were repeated three times.

### Western Blot Analysis

Total protein was extracted from BMDCs treated with PBS, KK2DP7 (80 µg mL^−1^), C29 (150 mµ for 2 h) + KK2DP7 (80 µg mL^−1^), or QNZ (10 nm for 2 h) + KK2DP7 (80 µg mL^−1^) for 24 h. The protein lysates (30 µg) were subjected to sodium dodecyl sulfate‒polyacrylamide gel electrophoresis and transferred to membranes（FastPAGE Precast Gel, Tsingke Biotechnology Co.,Ltd）. Then, the membranes were probed with antibodies against GAPDH (D16H11), NF‐*κ*B p65 (D14E12), and phospho‐NF‐*κ*B p65 (Ser536) (93H1) (CST, USA) and incubated with horseradish peroxidase (HRP)‐conjugated secondary antibody (Abcam, USA). Finally, a chemiluminescence system (Millipore, Massachusetts, USA) was used to visualize and photograph the target protein bands.

### Flow Cytometry Assay of the Lymph Nodes

In the in vivo experiment, to detect DC uptake efficiency in the LNs, Cy3‐OVA (20 µg), or KK2DP7 (60 µg)/Cy3‐OVA (20 µg) was injected subcutaneously near the right of the LNs of the mice. After 4 h, the right LNs were removed to evaluate the proportions of DC uptake of Cy3‐OVA by flow cytometry. To detect DC function and DC classification in the LNs, OVA (20 µg), DP7 (60 µg)/OVA (20 µg), DP7‐C (60 µg)/OVA (20 µg), KDP7 (60 µg)/OVA (20 µg), KK2DP7 (60 µg)/OVA (20 µg), KK2K4DP7 (60 µg)/OVA (20 µg), CpG (Invitrogen, 20 µg)/OVA (20 µg), poly (I:C) (Invitrogen, 50 µg)/OVA (20 µg), and Imject Alum (Thermo, 100 µg)/OVA (20 µg) were injected subcutaneously near the right of the LNs of the mice. After 24 h, the right LNs were removed to evaluate the proportions of mature DCs (CD80 (16‐10A1)^+^ CD86 (GL1)^+^ DCs), CD103 (M290)^+^ DCs, SIINFEKL‐H2K^b+^ DCs, cDC1s (CD8 (53‐6.7)^+^ DCs), cDC2s (CD4 (RM4‐5)^+^ DCs), pDCs, Mo‐DCs, and LCs (Langerhans cells) by flow cytometry. Flow cytometry was performed on a FACSAria SOPR (BD, USA) flow cytometer, and the data were analyzed with FlowJo 10.8.1 software.

### Prophylactic and Therapeutic Studies with OVA‐Based Nanovaccines

For the prophylactic study, different vaccines were subcutaneously injected near the right of the paralymph node in the female C57BL/6 mice from each group three times at 1‐week intervals. 7 days after the final vaccination, the vaccinated mice were subcutaneously challenged on the right back with 1 × 10^6^ EG7‐OVA cells. The tumor volume was measured with a caliper every other day and calculated according to the following formula: V = length × width × width/2 (mm^3^). For the therapeutic study, female C57BL/6 mice were subcutaneously injected on the right back with 1 × 10^6^ EG7‐OVA cells and were subcutaneously vaccinated with different kinds of vaccines, as described in the main text, on days 4, 11, and 18. The tumor volume and body weight of the mice were recorded every 2 days. Tumor volume = 4/3 × *π* × length/2 × width/2 × width/2. Mice were euthanized when the tumor volumes reached 1500 mm^3^.

### T‐Cell Proliferation Assay

To verify the effect of the KK2DP7/OVA vaccine on T‐cell proliferation, splenocytes from mice immunized with KK2DP7 (60 µg)/OVA (20 µg) three times (once a week) were separated on day 7 post‐immunization using a lymphocyte isolation solution (Dakewe Biotech Co., Ltd.) and stained with a CFSE kit (Beyotime Biotechnology) according to the manufacturer's protocol. The CFSE‐stained splenocytes were stimulated with OVA for 3 days and then stained with anti‐mouse CD3 (145‐2C11) and anti‐mouse CD8 (53‐6.7) fluorescent antibodies (BD). Then, cell proliferation was measured by flow cytometry.

### CD8 Tetramer Detection

Splenocytes from mice immunized with KK2DP7 (60 µg)/OVA (20 µg) three times (once a week) were separated on day 7 post‐immunization and stained with phycoerythrin‐labeled SIINFEKL‐MHC I tetramer. Anti‐CD8‐APC (53‐6.7) and anti‐CD3‐FITC (145‐2C11) were analyzed to determine the percentages of OVA‐specific CD8^+^ T cells using a tetramer staining assay following the standard protocol.

### ELISPOT Assay

For the ELISPOT assay, splenocytes from mice immunized with KK2DP7 (60 µg)/OVA (20 µg) three times (once a week) were separated for experiments on day 7 post‐immunization. The ELISPOT assay was performed according to the manufacturer's instructions. Briefly, 2 × 10^5^ mouse spleen lymphocytes were seeded in a 96‐well microtiter plate precoated with an anti‐IFN‐*γ* antibody. Next, 10 µg mL^−1^ OVA was added, and the plate was incubated at 37 °C. After 48 h, the culture medium was aspirated from the wells, and precooled ddH_2_O was added at 4 °C for 10 min to lyse the cells. The plate was then washed five times with wash buffer. Next, a diluted biotinylated secondary antibody was added to each well, followed by incubation for 1 h at 37 °C. For enzyme‐linked avidin incubation, a diluted avidin enzyme working solution was added to each well and incubated at 37 °C for 1 h. A prepared aminoethyl carbazole solution was then added, and the color reaction was allowed to occur at 37 °C in the dark for ≈15 min. Finally, the plates were photographed and read using a BioReader 4000 (Byosys, Karben, Germany).

### Tumor Recurrence Prevention Model with OVA‐Based Nanovaccines

For this study, the female C57BL/6 mice were randomly divided into four groups: 1) surgery (*n* = 6), 2) surgery+anti‐PD‐1 antibody (*n* = 6), 3) surgery+KK2DP7/OVA (*n* = 6), and 4) surgery+KK2DP7/OVA+anti‐PD‐1 antibody (*n* = 6). On day 10, mice were injected subcutaneously on the right back with 1 × 10^6^ EG7‐OVA cells. After 10 days, subcutaneous tumors with a volume of ≈150 mm^3^ were resected. On days 4 and 11, mice in groups 3 and 4 were vaccinated with KK2DP7 (60 µg)/OVA (20 µg) by subcutaneous injection. Mice in groups 2 and 4 were administered an anti‐PD‐1 antibody (200 µg per mouse for each injection) intraperitoneally on days 5, 8, 12, and 15. The tumor volume and body weight of the mice were recorded every 2 days. Tumor volume = 4/3 × *π* × length/2 × width/2 × width/2. Mice were euthanized when the tumor volumes reached 1500 mm^3^. On day 40, the mice that survived were subcutaneously injected with 1 × 10^6^ EG7‐OVA and CT26 cells to evaluate immunological memory. For the tumor‐free mice that survived to 60 days after EG7‐OVA cell challenge (KK2DP7/OVA+anti‐PD‐1), spleen cells were harvested from the surviving mice and stained with anti‐CD3 (145‐2C11), anti‐CD8 (53‐6.7), anti‐CD62L (MEL‐14), and anti‐CD44 (IM7). Data analysis was carried out using FlowJo software.

### Therapeutic Studies with Neoantigen‐Based Nanovaccines

For this study, female C57BL/6 mice were randomly divided into four groups: 1) PBS (*n* = 7), 2) neoantigen (*n* = 7), 3) KK2DP7/neoantigen (*n* = 7), and 4) Freund's adjuvant/neoantigen (*n* = 7). On day 0, mice were intravenously injected with 5 × 10^5^ LL2 cells. On days 1, 8, and 15, mice in group 2 were vaccinated with neoantigen (100 µg) by subcutaneous injection, and mice in group 3 were vaccinated with KK2DP7 (60 µg)/neoantigen (100 µg) by subcutaneous injection. On day 1, mice in group 4 were vaccinated with complete Freund's adjuvant + neoantigen (100 µg) by subcutaneous injection. On days 8 and 15, mice in group 4 were vaccinated with incomplete Freund's adjuvant + neoantigen (100 µg) by subcutaneous injection. Lung weights and the number of lung nodules will be measured once the mice have passed away.

### Histological Analysis

After the mice in each group were sacrificed, the main organs were harvested and fixed immediately using 4% paraformaldehyde for 72 h. Then, the tissues were cut into thin slices of no more than 5 mm and placed in the embedding cassette, which was rinsed overnight using tap water. The subsequent tissue dehydration and embedding process was as follows: 1) The embedding cassette with the tissue block was transferred to 75% ethanol and soaked overnight. 2) It was transferred to 85% ethanol and soaked for 30 min. 3) It was transferred to 95% ethanol and soaked twice for 15 min each time. 4) It was transferred to 100% ethanol and soaked three times for 15 min each time. 5) They were transferred to xylene solution and soaked twice for 15 min. 6) They were transferred to fresh paraffin solution three times for 30, 20, and 10 min. 7) They were transferred to fresh paraffin for embedding. Finally, the embedded tissue sections were dewaxed and rehydrated before staining with Mayer's hematoxylin and eosin (H&E) according to the vendor's instructions (Solarbio, China).

### Statistical analysis

All the values in the present study are presented as the mean ± s.d. unless otherwise indicated in the figure captions. One‐way analysis of variance was used for multiple comparisons (ANOVA) when more than two groups were compared, followed by post hoc Tukey's multiple comparison test, and Student's *t*‐test was used for two‐group comparisons. All the statistical analyses were carried out with the GraphPad Prism software package (PRISM 8.0; GraphPad Prism Software). Survival curves were obtained using the Kaplan–Meier method and compared by the log‐rank test. The threshold for statistical significance was *p* < 0.05. * indicates *p* < 0.05, ** indicates *p* < 0.01, *** indicates *p* < 0.001, **** indicates *p* <  0.0001.

## Conflict of Interest

The authors declare no conflict of interest.

## Author Contributions

Y.L. and Z.R. designed the study, Z.R. responsible for all experiments and articles; T.L. and W.Y.S. helped ZR to perform the tumor model and the in vitro experiment; T.L., T.Y.M., Z.B.Y., Z.B.L., W.S.W., X.D.Y., and Y.Y.L. helped Z.R. to perform the date analysis. All authors read and approved the final manuscript.

## Supporting information

Supporting Information

## Data Availability

The data that support the findings of this study are available from the corresponding author upon reasonable request.
