# Supplementary material for: A Dendrimer Peptide (KK2DP7) Delivery System with Dual Functions of Lymph Node Targeting and Immune Adjuvants as a General Strategy for Cancer Immunotherapy
Source: Adv Sci (Weinh). 2023 Mar 22;10(15):2300116. doi: 10.1002/advs.202300116 (PMC10214225; doi:10.1002/advs.202300116)
Supplement: Supplementary file 1 — Supporting Information [file ADVS-10-2300116-s001.pdf]

ADVANCED SCIENCE
Open Access

Supporting Information

for *Adv. Sci.*, DOI 10.1002/advs.202300116

A Dendrimer Peptide (KK2DP7) Delivery System with Dual Functions of Lymph Node Targeting and Immune Adjuvants as a General Strategy for Cancer Immunotherapy

*Rui Zhang, Lin Tang, Yusi Wang, Yaomei Tian, Siwen Wu, Bailing Zhou, Chunyan Dong, Binyan Zhao, Yuling Yang, Daoyuan Xie and Li Yang**

Supplementary materials

Materials and methods

Flow cytometry assay of the lymph nodes (LNs)

To examine macrophage function and classification in the LNs, OVA (20 μg), KK2DP7 (60 μg)/OVA (20 μg), CpG (20 μg)/OVA (20 μg), poly(I:C) (50 μg)/OVA (20 μg), and Imject® Alum (100 μg)/OVA (20 μg) were injected subcutaneously near the LNs in mice. After 24 hours, the LNs were removed to assess the proportions of total macrophages (CD11b$^+$ F4/80$^+$ cells), M1 macrophages (CD86$^+$), and M2 macrophages (CD206$^+$) by flow cytometry.

In vitro cytotoxicity assessment

The cytotoxicity of KDP7, KK2DP7 and KK2K4DP7 was measured via a well-established CCK-8 assay. Generally, DCs incubated with KDP7, KK2DP7 and KK2K4DP7 at various concentrations were seeded in a 96-well plate at a density of approximately 5×10^4 cells for 24 h. Then, a standard CCK-8 assay was used to determine the relative cell viability.

Lymph node accumulation of the peptide/OVA$_{257-264}$ complex and peptide/OVA$_{257-264}$ complex-treated DCs

To detect the accumulation of the peptide/OVA$_{257-274}$ complex in lymph nodes after subcutaneous injection, Cy5-OVA$_{257-264}$ (20 μg) and DP7 (60 μg), DP7-C (60 μg), KDP7 (60 μg), KK2DP7 (60 μg), and KK2K4DP7 (60 μg) were coincubated and then injected subcutaneously near the lymph nodes. The lymph nodes at the injection sites were removed after 4 h, and the presence of fluorescence in the lymph nodes was detected by ex vivo imaging using a PerkinElmer IVIS Lumina III.

To detect the accumulation of peptide/OVA$_{257-264}$ complex-treated DCs in the lymph nodes after subcutaneous injection, OVA$_{257-264}$ (10 μg/ml) and DP7 (25 μg/ml), DP7-C (25 μg/ml), KDP7 (25 μg/ml), KK2DP7 (25 μg/ml), and KK2K4DP7 (25 μg/ml) were coincubated for 10 min and then added to DCs for 24 h. Next, the DCs were labeled with Dir and then injected subcutaneously (1×10^6 cells/per mouse) near the lymph nodes. All of the lymph nodes at the injection sites were removed after 24 h, and the presence of fluorescence in the lymph nodes was detected by ex vivo imaging using a PerkinElmer IVIS Lumina III.

Molecular docking of KK2DP7 and TLR2

Molecular docking was conducted in MOE v2018.0101. The 2D structure of KK2DP7 was prepared by molecule and protein build module in MOE and converted to 3D structure through energy minimization. The X-ray structure of protein TLR2 were downloaded from RCSB Protein Data Bank (PDB ID: 1O77). KK2DP7 was chosen as ligand and proteins as receptor. The binding site of protein TLR2 was found by Site Finder module in MOE, the top ranked one was used as binding site. Prior to docking, the force field of AMBER10:EHT and the implicit solvation model of Reaction Field (R-field) were selected. The docking workflow followed the “induced fit” protocol, in

which the side chains of the receptor pocket were allowed to move according to ligand conformations, with a constraint on their positions. The weight used for tethering side chain atoms to their original positions was 10. For the ligand, all docked poses of which were ranked by London dG scoring first, then a force field refinement was carried out on the top 30 poses followed by a rescoring of GBVI/WSA dG. The final best ranked pose was selected and for further all-atom, explicit water molecular dynamics simulation.

Molecular dynamics (MD) simulation of KK2DP7 and TLR2

The complexes of KK2DP7 with TLR2 after docking were optimized by MD simulation respectively. MMFF94x Force Field parameters were used for peptide KK2DP7. Hydrogen atoms of KK2DP7 were optimized by Gaussian09 package at the level of HF/6-31g*. Then the partial atomic charges were calculated by the restrained electrostatic potential (RESP) charge from the calculation with Gaussian09 package at HF/6-31g* level. Then the complex was then neutralized by adding sodium/chlorine counter ions and solvated in a cuboid box of TIP3P water molecules with solvent layers 10 Å between the box edges and solute surface.

All MD simulations were performed using AMBER16. The AMBER GAFF and FF14SB force fields were applied and the SHAKE algorithm was used to restrict all covalent bonds involving hydrogen atoms with a time step of 2 fs. The Particle mesh Ewald (PME) method was employed to treat long-range electrostatic interactions. For each solvated system, two steps of minimization were performed before the heating step. The first 4000 cycles of minimization were performed with all heavy atoms restrained with 50 kcal/(mol·Å2), whereas solvent molecules and hydrogen atoms were free to move. Then, non-restrained minimization was carried out involving 2,000 cycles of steepest descent minimization and 2,000 cycles of conjugated gradient minimization. Afterwards, the whole system was first heated from 0 K to 300 K in 50 ps using Langevin dynamics at a constant volume and, then, equilibrated for 400 ps at a constant pressure of 1 atm. A weak constraint of 10 kcal/ (mol·Å2) was used to restrain all the heavy atoms during the heating steps. Periodic boundary dynamics simulations were carried out for the whole system with an NPT (constant composition, pressure, and temperature) ensemble at a constant pressure of 1 atm and 300 K in the production step. In production phase, 100 ns simulation was carried out. The binding free energy of complex was calculated using the MM-GBSA method.

Results

Model of aggregation of the peptide monomer

The radius of gyration (Rg)[8] was first calculated to evaluate the aggregation of the four branches of the peptide monomer. The Rg is an important property of a structure that shows the shape of a molecule, and a larger Rg indicates more expansion of the molecule. As shown in Supplementary Figure 2a, the Rg values gradually decreased along with the simulation time after 120 ns and stabilized after 350 ns. The peptide monomer structure clearly started self-aggregating after 120 ns and was completely self-aggregated after 350 ns. The structures at different simulation times (0 ns, 100 ns,

200 ns, 300 ns, 400 ns and 477 ns), which are depicted in Figure 2b, confirm the variation tendency of the Rg values. This result indicated that along with the simulation time, the four branches of the peptide monomer gradually aggregated, and at the end of the simulation, the four branches of the peptide monomer were intertwined.

Model of aggregation of multiple peptides

Additionally, the radius of gyration (Rg) was first calculated to evaluate the model of aggregation of multiple peptides. As shown in Supplementary Figure 2b, in contrast to self-aggregation of the peptide monomer, which began to occur after only a short simulation time (120 ns), the Rg values of the multiple peptide model gradually decreased with the simulation time after 750 ns and stabilized after 900 ns, indicating that the aggregation of ten monomers should occur over a long simulation time (750 ns), with complete aggregation after 900 ns. The structures at different simulation times (0 ns, 200 ns, 400 ns, 600 ns, 800 ns and 985 ns), which are depicted in Figure 2c, confirm the variation tendency of the Rg values. This result indicated that after a long adjustment period, multiple peptide monomers could gradually aggregate, and at the end of the simulation, the four branches of the multiple peptide monomers were intertwined.

MD simulation result of KK2DP7 and TLR2

To search for the stable complex structure of KK2DP7 with TLR2, we performed a molecular docking search followed by all-atom, explicit water MD simulations. The backbone of Val1.A in KK2DP7 forms hydrogen bond interaction with amino group of Lys698 in TLR2. The Indolylamine of Trp3.A in KK2DP7 forms Arene-H interaction with amide group of Asn729 in TLR2. The backbone of Val1.A in KK2DP7 forms hydrogen bond interaction with hydroxyl group of Ser784 in TLR2. The guanidine group of Arg4.B in KK2DP7 forms salt bridges with carboxyl group of Glu727 in TLR2. The guanidine group of Arg11.B in KK2DP7 forms hydrogen bond interaction with amide group of Asn728 in TLR2. The guanidine group of Arg6.C in KK2DP7 forms salt bridge with carboxyl group of Glu741 in TLR2. The backbone of Val7.C in KK2DP7 forms hydrogen bond interaction with side chain of Lys759 in TLR2. The guanidine group of Arg6.C in KK2DP7 forms salt bridge with carboxyl group of Glu763 in TLR2. The backbone of Gln2.C in KK2DP7 forms hydrogen bond interaction with backbone of Met766 in TLR2. The backbone of Val9.C in KK2DP7 forms hydrogen bond interaction with backbone of Thr758 in TLR2.

The binding energy (ΔG_{total}) of KK2DP7 with TLR2 was calculated using the MM-GBSA method was shown in Table 1. The contribution to the binding free energy (ΔG_{total}) from the VdW and electrostatic interactions was represented by ΔE_{vdw} and ΔE_{elec}. The polar and nonpolar solvation energy contributions to ΔG_{total} were represented by ΔG_{polar} and $\Delta G_{nonpolar}$ respectively. The KK2DP7-TLR2 binding was largely governed by electrostatic interactions, with ΔE_{elec} being the most favorable contributor. ΔG_{polar} was unfavorable for the binding, while $\Delta G_{nonpolar}$ was favorable, which lead to an overall favorable binding energy. The binding free energy upon

KK2DP7 with TLR2 is computed to be −80.55 kcal/mol in aqueous environments.

Figures and figure legends

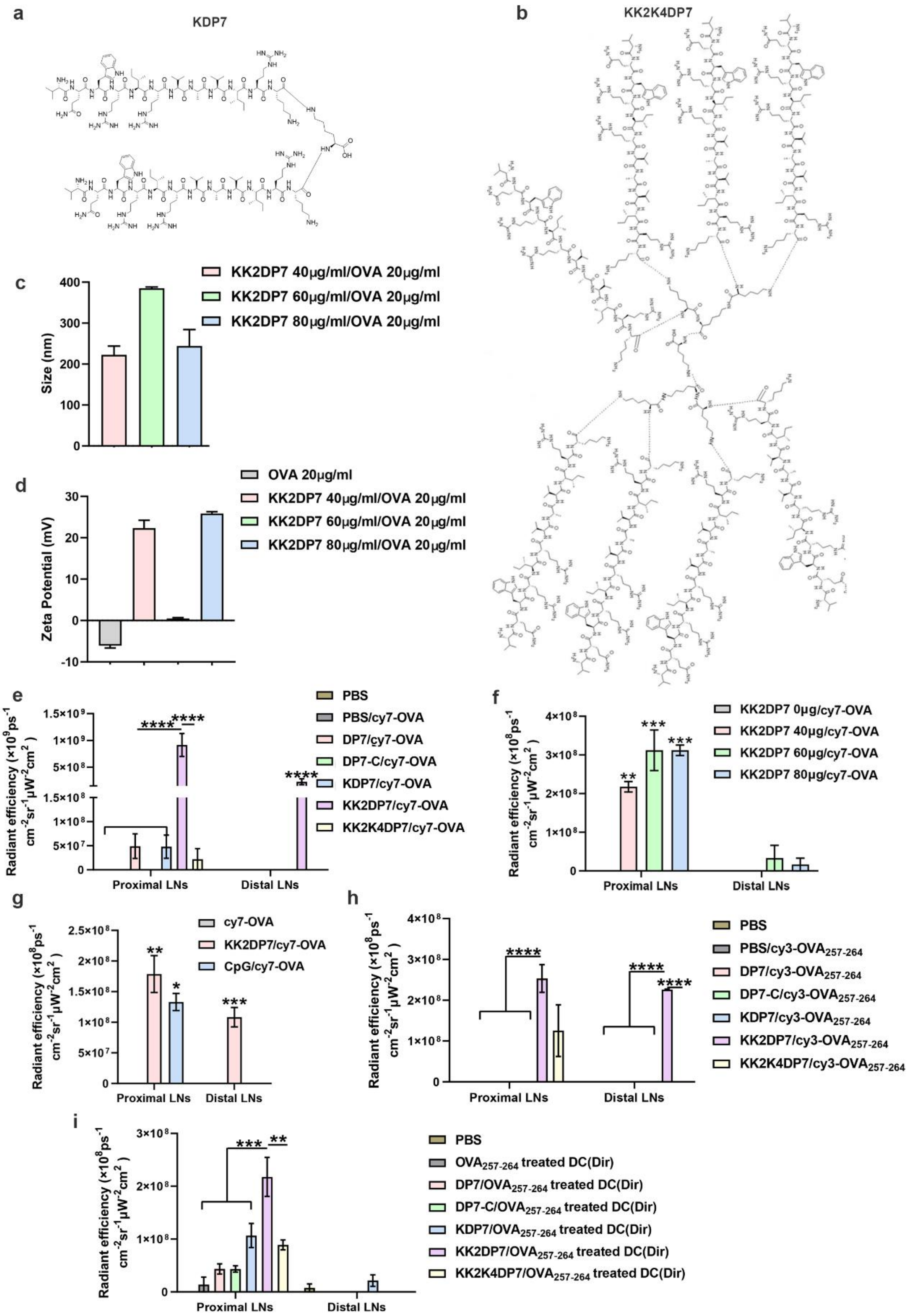

Supplementary Figure 1. Structure and sequence of DP7 series. a-b) The structural formulas of KDP7 and KK2K4DP7**.** c-d) Diameter and zeta potential of KK2DP7 (40, 60, 80 μg/ml)/OVA (20 μg/ml) (n = 3). e-i) Statistical results of Figure 1d-Figure 1h.

*$P < 0.05$, **$P < 0.01$, ***$P < 0.001$, ****$P < 0.0001$.

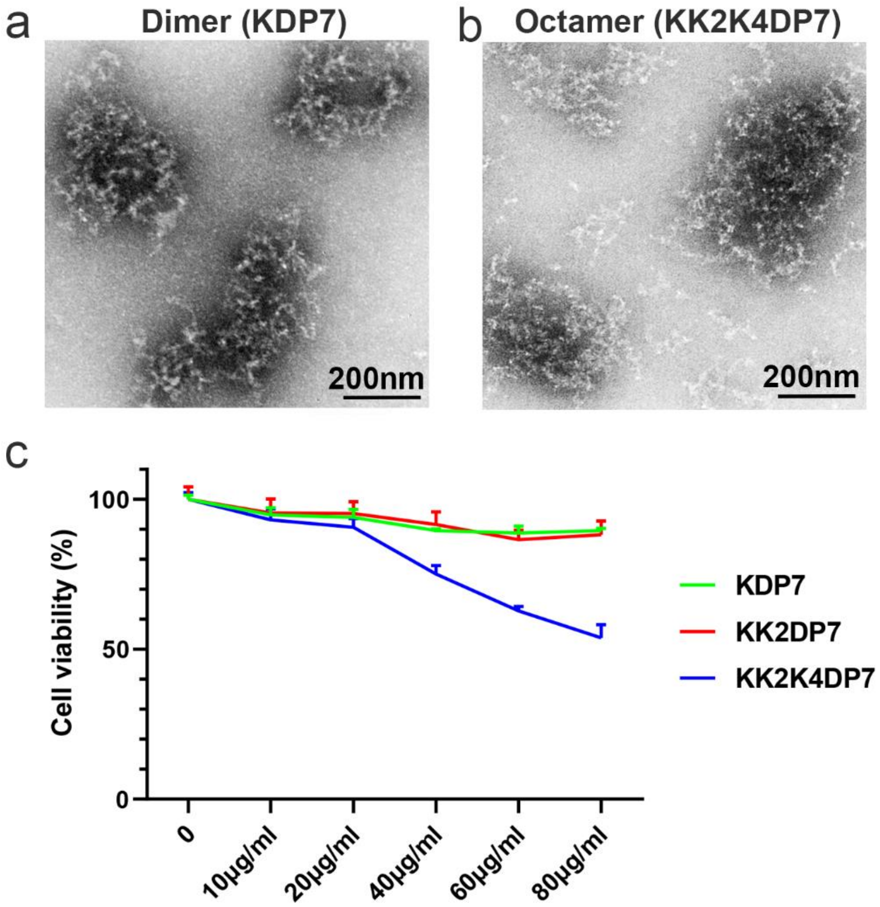

Supplementary Figure 2. a-b) TEM images of KDP7 and KK2K4DP7. c) CCK-8 assay of KDP7-(IC_{50}=430.3μg/ml), KK2DP7-(IC_{50}=538.6μg/ml) and KK2K4DP7-(IC_{50}=88.71μg/ml) treated DCs (n=3).

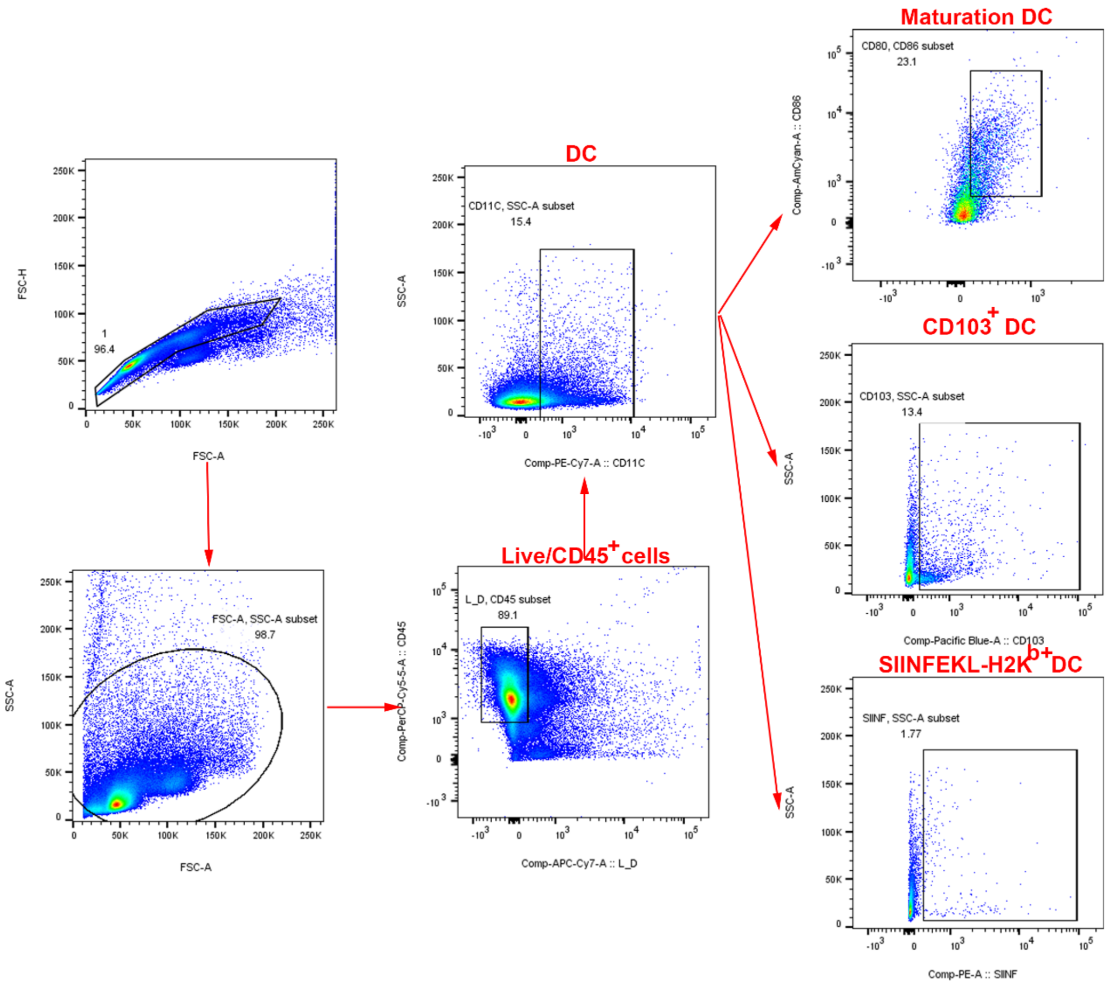

Supplementary Figure 3. Flow cytometry gating strategy for the analysis of DC function in LNs.

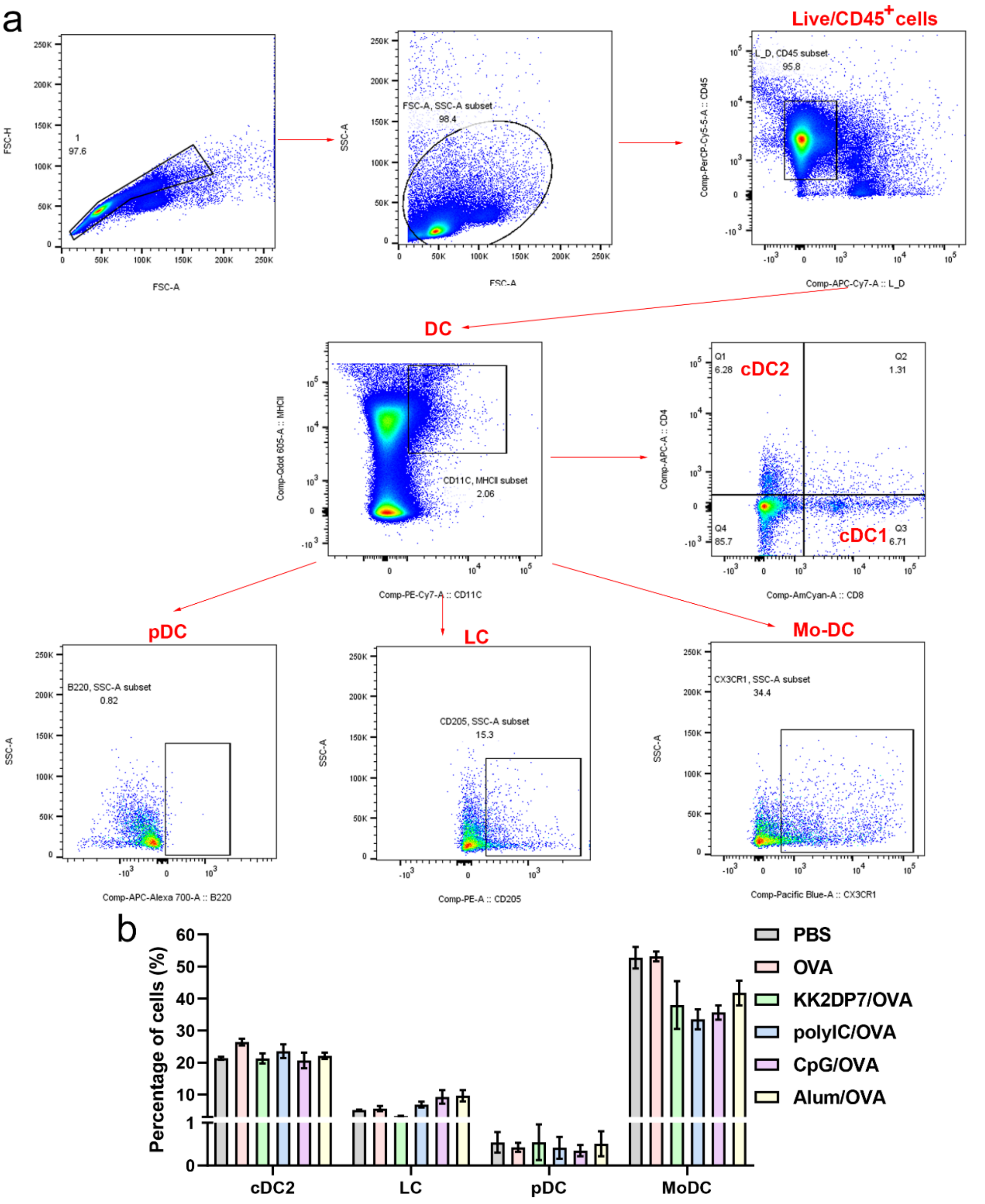

Supplementary Figure 4. a) Flow cytometry gating strategy for the analysis of DC classification in LNs. b) Percentages of cDC2s, LCs, pDCs, and MoDCs after different treatments (n=3).

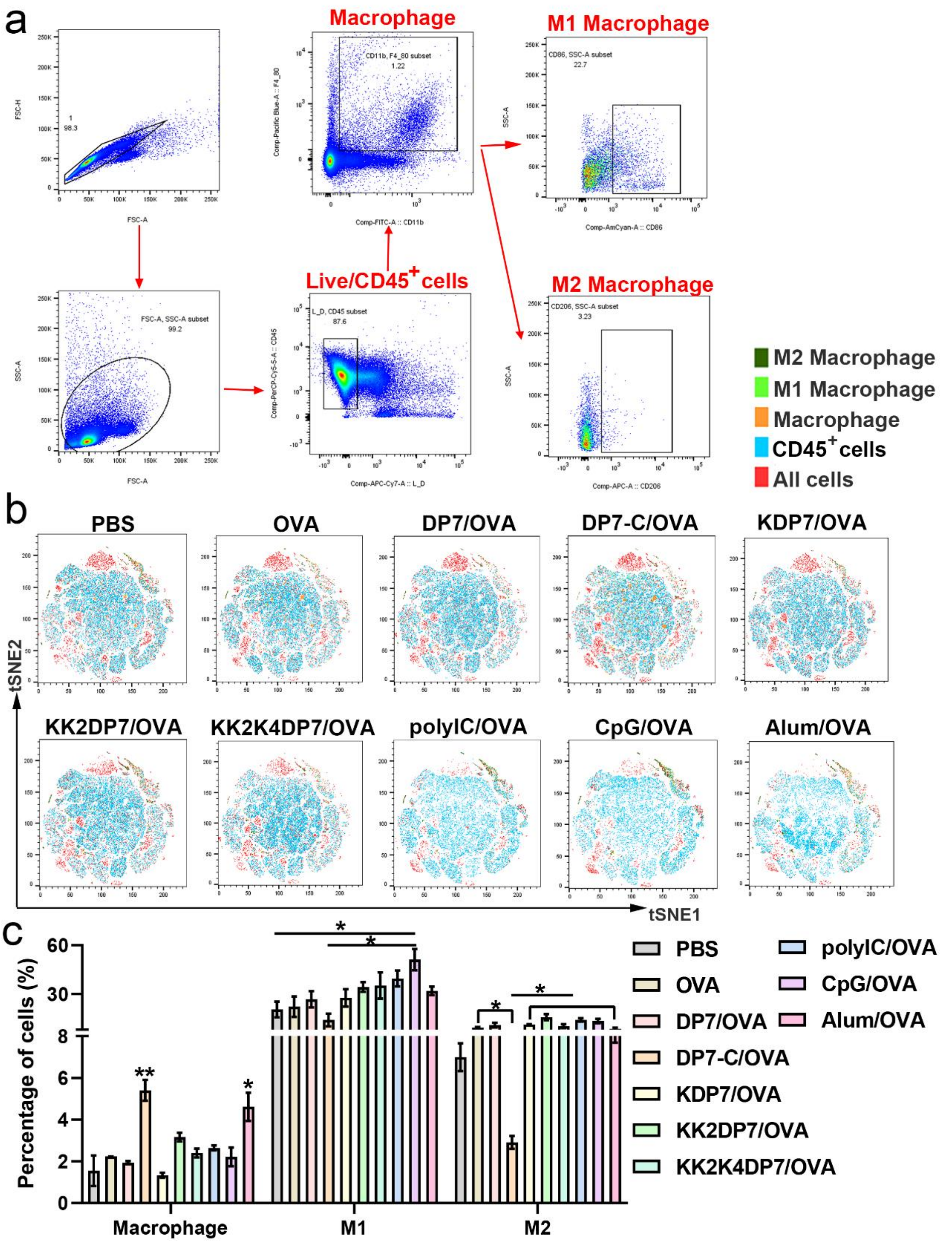

Supplementary Figure 5. a) Flow cytometry gating strategy for the analysis of macrophages in LNs. b) tSNE map of macrophages in LNs. c) Percentages of total macrophages, M1 macrophages, and M2 macrophages after different treatments (n=3). *$P < 0.05$, **$P < 0.01$.

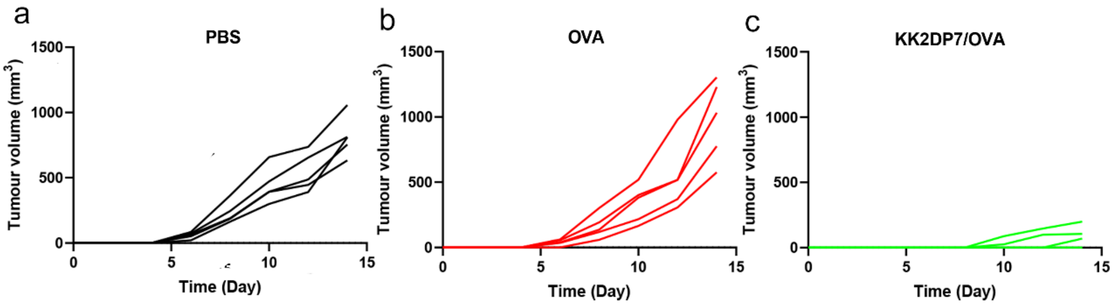

Supplementary Figure 6. a-c) Individual tumor growth curves from Figure 6e.

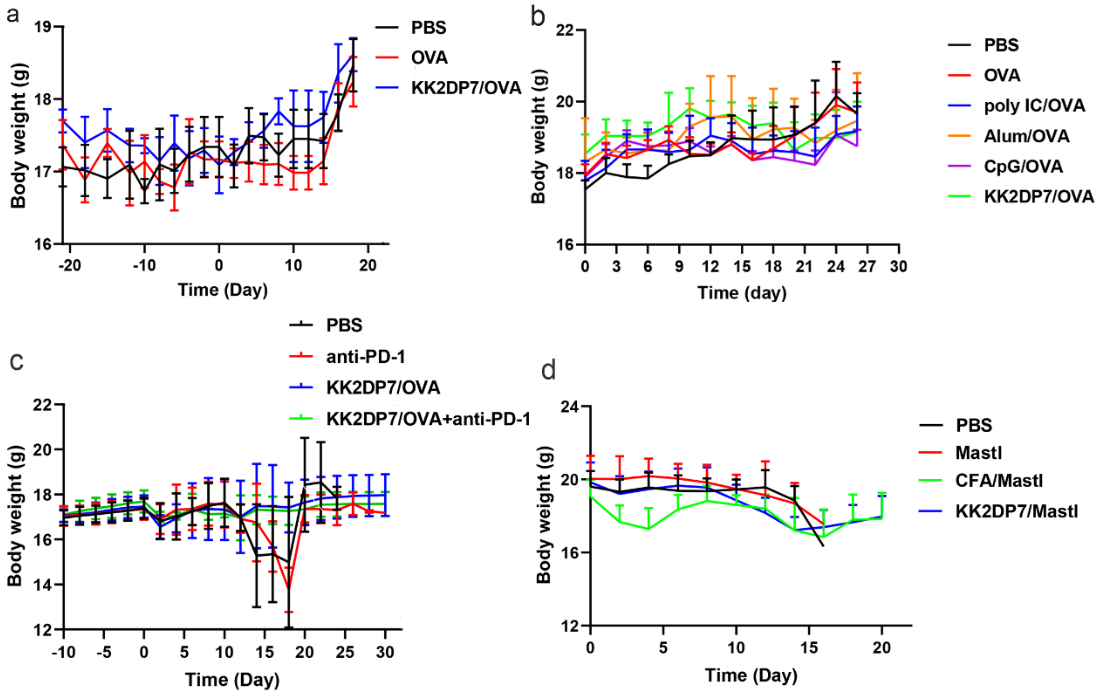

Supplementary Figure 7. a) Body weight of mice in Figure 5i. b) Body weight of mice in Figure 6b. c) Body weight of mice in Figure 6f. c) Body weight of mice in Figure 6l.

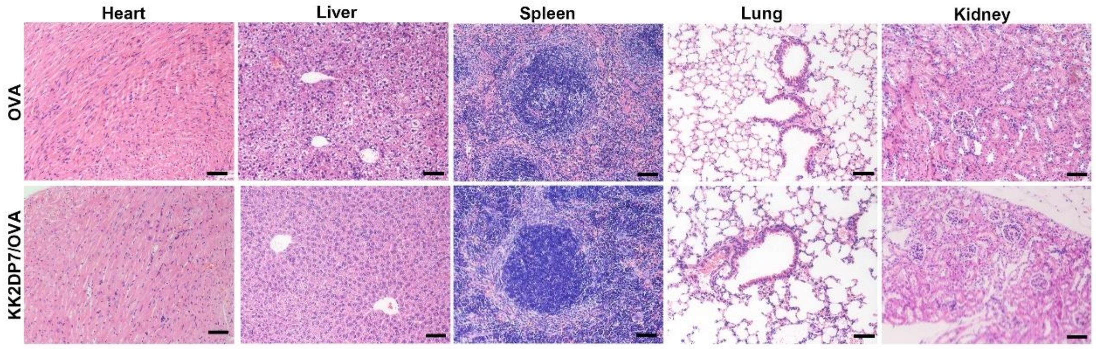

Supplementary Figure 8. H&E staining of major organs collected from mice 7 days after three intradermal injections of KK2DP7/OVA. No noticeable signs of organ damage appeared in any major organs of the mice. Scale bar, 50 μm.

Supplementary Table 1. Average Binding Energy and its Components Obtained from the MM-GBSA Calculation for the KK2DP7/TLR2 complex.

Contribution	Energy (kcal/mol)
ΔE_{vdw}	-93.32±1.33
ΔE_{elec}	-594.78±6.83
ΔG_{polar}	621.81±6.41
$\Delta G_{nonpolar}$	-14.26±0.17
ΔG_{total}	-80.55±1.22
